# Therapeutic potential of plant-based therapies in pediculosis capitis: Systematic review and meta-analysis

**DOI:** 10.1371/journal.pgph.0004841

**Published:** 2025-07-17

**Authors:** Jacqueline Chen, Angela Mei, Angela Jacques, Bernadette Ricciardo, Asha Bowen

**Affiliations:** 1 Department of Dermatology, Sir Charles Gairdner Hospital, Perth, Washington, Australia; 2 Department of Dermatology, Perth Children’s Hospital, Perth, Washington, Australia; 3 Institute for Health Research, The University of Notre Dame Australia and Department of Research, Sir Charles Gairdner Hospital, Perth, Washington, Australia; 4 Wesfarmers Centre for Vaccines and Infectious Diseases, Telethon Kids Institute, Perth, Washington, Australia; 5 Division of Paediatrics, School of Medicine, University of Western Australia, Perth, Washington, Australia; 6 Department of Dermatology, Fiona Stanley Hospital, Perth, Washington, Australia; 7 Department of Infectious Diseases, Perth Children’s Hospital, Perth, Washington, Australia; Management and Science University, MALAYSIA

## Abstract

Pediculosis capitis is a worldwide prevalent public health issue, mostly involving children. Resistance has been increasingly identified with conventional treatments such as permethrin or malathion. We aimed to evaluate the therapeutic potential of plant-based therapies for pediculosis capitis. PubMed, MEDLINE, Embase, EmCare, Web of Science, Cochrane, and ScienceDirect were searched for studies. Google Scholar was used to identify relevant gray literature from inception until 30^th^ July 2023. Randomized controlled trials (RCTs) and non-randomized controlled trials (NRCTs) in English language evaluating a plant-based head lice treatment were considered for inclusion. This review was registered with the International Prospective Register of Systematic Reviews (CRD42023428674). Study characteristics, detection method, outcomes including final cure rate at 1–14 days following last treatment and adverse events were identified. Statistical analysis was performed with one sample t-test and linear mixed models. Random effects meta-analysis using forest-plots were used to describe intervention odds ratio. From 881 records, 20 studies were included comprising 13 RCTs and 7 NRCTs. All 20 studies were qualitatively analyzed and 9 RCTs were quantitatively analyzed. Based on RCT data, certain plant-based interventions may offer improved cure rates with overall higher mean final cure rate 0.86 (95% CI 0.73, 0.99) compared to conventional treatment 0.60 (95% CI 0.25, 0.95), however findings were limited by study heterogeneity (I^2^ = 83.2%) and methodological constraints. Local cutaneous irritation was the most frequent adverse event. Several limitations were identified, including confounding variables (e.g., inconsistent combing technique and variable plant-based interventions with multiple active ingredients and formulations), small sample sizes and lack of blinding. The risk of bias was high for NRCTs, while RCTs had some concerns. In conclusion, results should be interpreted cautiously in the context of study limitations. Further research is required to elucidate the efficacy and clinical role of plant-based therapies in PC.

## Introduction

Pediculosis capitis (PC), also known as head lice, is a pruritic infestation of the scalp caused by the insect *Pediculus humanus* capitis [1]. Predominantly affecting children, PC is the third most common cause of reported outbreaks in daycare centres and schools [2]; with female predilection (>2:1) [3].

PC prevalence varies globally, with higher rates observed in lower-income regions (e.g., 8.8% in Iran [4], 35% in Malaysia [5]) compared to high-income countries such as Australia (5.3%) and South Korea (4.1%) [6–8]. This disparity may be attributed to differences in hygiene practices such as low shower frequency and shared personal hygiene items [4]. Although PC disproportionately affects lower socioeconomic groups due to limited healthcare access and poor sanitation, it also persists in high-income countries, as head lice can spread through any social setting involving direct contact, such as schools or playdates. Rapid treatment access and improved hygiene mitigate but do not eliminate outbreaks. A large cross-sectional study in Poland (N = 95,153) found a higher PC prevalence in rural schools (1.59%) than in urban schools (0.48%), highlighting the impact of healthcare accessibility on infestation rates [9].

Within Australia, a high prevalence of PC has been described in both rural- and urban-living Aboriginal and/or Torres Strait Islander children (hereafter respectfully referred to as Aboriginal). The Koolungar Moorditj Healthy Skin project is the first co-designed Australian study to describe skin health and disease in urban-living Aboriginal children. Findings from the 2021 pilot study with 80 urban-living West Australian (WA) Aboriginal children (0–18 years) revealed a 23% (18/79) point prevalence of PC [10]. Among rural living Aboriginal children in WA, the ‘Healthy Skin Initiative’ reported a point prevalence of 21% (22/103) [11].

Scalp pruritus and irritation from PC can be debilitating, impacting on mental health and leading to anxiety, bullying and non-attendance at school and employment [1]. Scratching can lead to secondary bacterial skin infection with *Staphylococcus aureus* and *Streptococcus pyogenes*, as well as potentially serious complications including sepsis, bone/joint infections, post-streptococcal glomerulonephritis and rheumatic heart disease. There are several reported cases of iron deficiency anaemia associated with chronic blood loss due to longstanding PC [12,13].

PC treatment presents challenges due to increasing resistance to commonly used pediculicides, the need for repeated applications, and safety concerns, particularly in young children. Typical treatments include a combination of wet-combing and chemical pediculicides recommended for the individual and close contacts. Wet combing is the mechanical removal of lice and eggs and serves as an alternative to topical pediculicides, particularly for children under 2 years of age [14]. Other non-neurotoxic topical therapies include dimethicone 4% or benzyl alcohol 5%. They cause airway asphyxiation of the pediculus, and treatment is often repeated in 7–10 days as these are not ovicidal [15]. Conventional products include permethrin and other synthetic pyrethroids, pyrethrins derived from chrysanthemum, and malathion which is an organophosphate. These result in neuronal hyperstimulation and louse paralysis, also requiring reapplication after 7 days [16]. Spinosad is a fermentation product of the soil bacterium *Saccharopolyspora spinosa* that has been found to be effective against lice [17].

Permethrin resistance is prevalent, with therapeutic efficacy diminishing from almost 100% in the 1980s to as low as 25% today [18]. This may be associated with the recessive knockdown resistance (*kdr)* gene which is observed in variable frequencies across different geographical areas, ranging from 0.00 in Ecuador and South Korea, to 0.93 in France, and 0.97 in the United States of America (USA) [18]. Australia, England, Israel and Turkey have observed 100% *kdr* gene frequencies [19]. However, a recent German study demonstrated that despite the highly prevalent *kdr*-like gene, this did not conclusively correlate with permethrin treatment failure [18]. Malathion resistance has been reported in Australia, United Kingdom, France and Denmark. The primary mechanism of malathion resistance is thought to be due to raised esterases in headlice, which increase detoxification of the insecticide into an inactive metabolite [20].

Ivermectin, an anthelminthic agent, binds to glutamate-gated chloride channels (GluCl) in lice, inducing hyperpolarization, paralysis and subsequent death [18]. Topical ivermectin 0.5% lotion and 2 doses of oral ivermectin 200–400 mcg/kg/dose 7 days apart have been used in PC [18]. Two multi-site RCTs reported clinical efficacy of a single topical ivermectin lotion application of 94.9% at Day 2 and 73.8% at Day 15 [21]. The efficacy of a single dose of oral ivermectin at 200 mcg/kg/dose, with repeat dose if viable lice were observed, was 93% [22]. Two doses of oral ivermectin at 400 mcg/kg/dose a week apart demonstrated 97.1% efficacy [23]. Topical ivermectin is available in the USA but not currently in Australia. Additionally, oral ivermectin in Australia for PC can only be accessed via off-label prescription and is not licensed for use in patients under 5 years of age, under 15 kilograms, or those who are pregnant or breastfeeding. Due to high costs, AUD $60 for an oral ivermectin course and USD $275 for a topical ivermectin course, ivermectin is often a second to third line treatment [24].

Although ivermectin resistance to PC is infrequently observed, two cases have been reported in Senegal^,^ associated with A251V, S46P and H272R GluCl mutations [25]. There is also reported emerging clinical resistance to crusted scabies in patients who have received multiple doses of ivermectin [26]. Ivermectin is largely safe, however, pruritus, lymphadenitis, arthralgia and fever are documented potential adverse effects. Rare neurotoxicity can occur, manifesting as dizziness, seizures, and altered mentation. Bomze et al reported several cases of ivermectin-related severe cutaneous adverse reactions [27]. Although ivermectin has high efficacy, limitations include cost, accessibility, emerging resistance and adverse effects.

Despite the availability of conventional pediculicides such as permethrin and malathion, increasing resistance, cost barriers, and concerns about safety necessitate alternative treatment strategies. Plant-based treatments, including essential oils, neem, and coconut-derived compounds, have shown potential, but a systematic synthesis of their efficacy and safety remain unexplored. This review aims to critically synthesize the efficacy and safety of plant-based therapies for PC, providing a comparative analysis with conventional pediculicides.

## Methods

The study was conducted as per the Preferred Reporting Items for Systematic Reviews and Meta-Analyses Protocols (PRISMA). The study was registered with the International Prospective Register of Systematic Reviews (CRD42023428674), with protocol review conducted prior to data collection and priori protocol amendment (Version 2.0) to include meta-analysis to provide quantitative data synthesis. A revision of studies included was undertaken, based on reviewer feedback, to ensure strict predefined inclusion criteria were met with full details of post-review adjustments available in S2 File.

### Search strategy

A search from inception until 30^th^ July 2023 was conducted from databases (PubMed, Medline, Embase, Emcare, Web of Science, Cochrane and Science Direct). Google Scholar was searched for gray literature limited to the first 200 results. Reference lists of included articles were hand searched for additional manuscripts. Keywords were “head lice” OR headlice OR pediculus OR pediculosis OR “head louse” AND treatment OR management OR remedy OR pediculicide OR therapeutic AND plant OR traditional OR herb OR botanical OR natural OR ethnomedical OR “bush medicine”. Full search strategies are available in S1 File.

### Selection of studies

RCTs and NRCTs in English evaluating a plant-based head lice treatment were considered for inclusion. Exclusion criteria included non-English language studies due to limitations of translation resources, unclear intervention due to lack of explicit treatment protocol and not reporting outcomes of interest. Quantitative synthesis was conducted for RCTs reporting clinical cure rates as the primary outcome. The title and abstracts were screened for suitability, and the full article texts of the included studies were reviewed by two independent authors for eligibility based on inclusion and exclusion criteria, data extraction and risk of bias (RoB). Discrepancies between reviewers were resolved through discussion with a third reviewer. Hand-searched studies were screened and included based on the same predefined eligibility criteria as studies identified through electronic databases.

### Intervention(s)/ Comparator(s)

All treatments containing materials of plant origin were accepted, including combination treatments. Wholly synthetic compounds were excluded. All therapeutic comparators comprising insecticides and dimethicone were accepted, herein referred to as ‘conventional treatment’.

### Outcome(s)

The primary outcome was final cure rate at 1–14 days following last treatment, measured as the proportion of participants free of head lice. Secondary outcomes were initial cure rate at 1–7 days following last treatment, and adverse events.

### Data extraction

The following data were extracted [S1 Table]:

- General information and study characteristics: funding, journal, study design, participant sex, age and ethnicity.
- Detection method
- Intervention: country of origin of plant, part of plant used, active component(s), dose, frequency, duration and route of administration.
- Comparator: dose, duration and frequency
- Outcomes: initial and final cure rates, other efficacy outcomes, adverse events

### Statistical analysis

Controls were based on usual care (insecticides) and interventions were based on plant-based treatments which were grouped into three categories: n = 2 anise based, n = 3 Eucalyptus oil based (EO) and n = 6 Others (including neem, lavender and tea tree oil, *Peganum harmala, Protium hepatophyllum*). Cure rates of individual studies were described using proportions based on cure events/ total events for intervention and control groups, with intervention group cure rate ratios. Mean proportional cure rate differences were compared using one-sample t-tests (test values based on control means). Studies with repeated follow ups at 7 and 14 days were examined using linear mixed models with results summarised as mean estimates and mean differences with 95% confidence intervals. Random effects meta-analysis was used to graphically describe (using forest plots) intervention odds of cure compared to usual care, with results summarised as pooled odds ratios and 95% confidence intervals. Studies missing data were not included in statistical analysis. Stata version 18.0 (StataCorp, College Station, TX) was used for data analysis.

### Risk of bias

The Cochrane RoB-2 tool was used to determine risk of bias for RCTs, to assess the intention to treat effect [28]. A modified Cochrane ROBINS-I tool was used for NRCTs, omitting questions relating to comparator groups for single arm trials [29].

## Results

### Study selection

**Fig 1:**
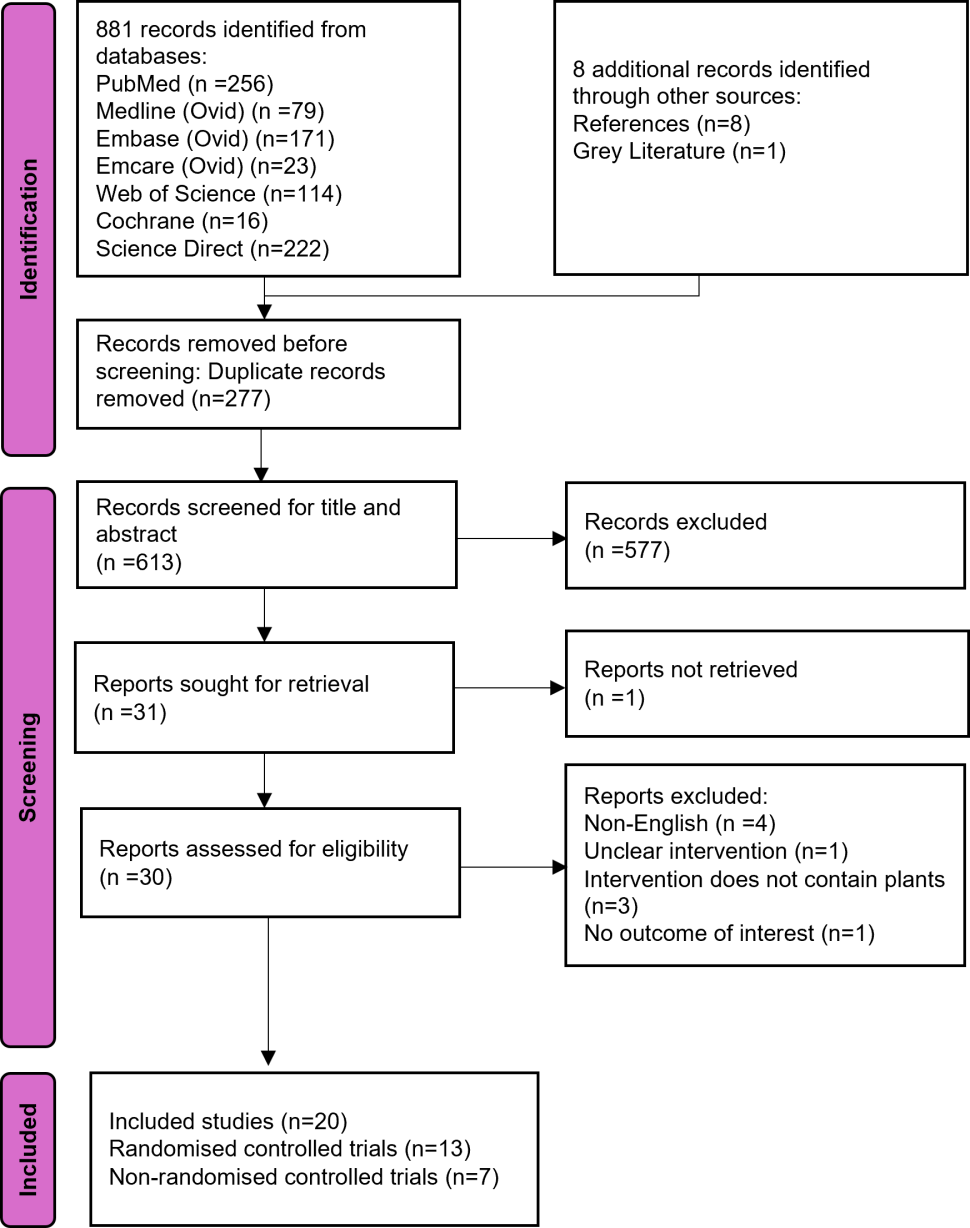
Flowchart for systematic review according to the PRISMA 2020 statement.

Our search yielded 890 records; 277 duplicates were removed. 613 records were screened based on title and abstract and 577 were excluded. Thirty-one records were retrieved for full text, with one unable to be retrieved. Thirty records were assessed for eligibility: 4 non-English studies were excluded, 1 excluded as it measured prevention and not efficacy outcomes, 3 excluded as did not contain plant material, 1 excluded due to plant being unspecified, and 1 excluded due to comparison of combing methods with plants in both intervention and comparator. In particular, 2 studies with fractionated coconut oil which contains only synthetic caprylic capric triglyceride were excluded. Twenty studies (13 RCTs [30–42] [Table 1] and 7 NRCTs [43–49] [Table 2] were included in the qualitative synthesis. Among these, 9 RCTs were included in the quantitative synthesis. (Fig 1)

**Table 1:** Summary of randomized controlled trials.

Author (year)	Study design	N=	Plant/s (Scientific)	Intervention	Dose, duration, frequency	Intervention final cure rate (%)	Adverse events	Comparator	Dose, duration, frequency	Comparator final cure rate (%), p-value	Adverse events
Cardoso et al. (2020)	RCT, double-blind	45	*Protium hepatophyllum*; in grape-seed oil and *Citrus aurantium*	Resin extract of *P. hepatophyllum* in grape seed oil, orange peel wax, surfactants (Eur-Amid N2)	1 treatment for 15min	21/22 (95.4%)	None	Permethrin 1% shampoo	1 treatment for 15 min	9/23 (39.1%, p < 0.0001)	None
Maarefvand et al. (2019)	RCT, non-blinded	93	*Peganum harmala* L.	*Peganum harmala* oil	6 treatments for 20min (D1,2,3 and 8,9,10)	44/48 (91.7%)	Irritation^a^/ headache: 1	Permethrin shampoo	2 treatments for unknown duration (D1 and D10)	39/45 (86.7%, p < 0.005)	Irritation^a^: 2 Headache: 1
Sabellina et al. (2018)^b^	RCT, single-blind	30	*Azadirachta indica*	Neem seed oil 10% methanolic shampoo	3 treatments for 10–15min (D0, D10, D20)	Mean reduction of headlice count: 17.8 + /- 23.97, p = 0.043	None	Permethrin 1% shampoo Pure shampoo (glycol stearate)	3 treatments for 10–15min (D0, D10, D20)	Mean reduction of head lice count: Permethrin: 22.5 + /- 23.47, p = 0.013 Pure shampoo: NR	None
Semmler et al. (2017)	RCT, single-blind	119	*Azadirachta indica*	Neem shampoo (Licener)	2 treatments for 10min (D1, D9)	60/60 (100%)	None	Dimethicone (Jacutin)	2 treatments for 10 min (D1, D9)	52/54 (96.2%, p = 0.0024)	None
Greive and Barnes, (2017)	RCT, single-blind	97	*Eucalyptus*, *Leptospermum petersonii*	Eucalyptus oil 11% and Leptospermum petersonii 1% (MOOV HeadLice solution)	3 treatments for 10min (D0, D7, D21)	33/40 (82.5%)	Irritation^a^: 18	Pyrethin 1.65mg/g/ Piperonyl Butoxide 16.5 mg/g (Banlice Mousse)	2 treatments for 10 min (D0, D7)	13/36 (36.1%, p < 0.001)	Irritation^a^: 3
Greive and Altman (2007)	RCT, double-blind	113	*Eucalyptus, Leptospermum petersonii*	Eucalyptus oil 11%, Leptospermum petersonii 1% (MOOV HeadLice Solution)	3 treatments for 10min (D0, D7, D14)	33/40 (82.5%)	Irritation^a^: 18	16.5mg/g Piperonyl butoxide and 1.65mg/g pyrethin (BanLice Mousse) 1% malathion (KP24)	2 treatments for 10 minutes (BanLice) or 30 min (KP24) (D0, D7)	Banlice 13/36 (36.1%, p < 0.0001) KP24 11/37 (26.7%, p < 0.0001)	Irritation^a^: 3
Mumcuoglu et al. (2002)	RCT, non-blinded	143	*Cananga odorata*, *Cocos nucifera*, *Pimpinella anisum*	Coconut, anise and ylang ylang oil (Chick-Chack spray)	3 treatments for 15min (D1, D5, D10)	60/70 (85.7%)	Irritation^a^: 1 Odour: 5	Permethrin 0.5%, Malathion 0.25%, Piperonyl butoxide 2%, Isododecane 47.25% and propellant gas 50% (Paraplus)	2 treatments for 10 min (D1, D10)	56/73 (56.7%, p > 0.05)	Irritation^a^:1 Odour: 4
Moreno-Alsasua (2016)	RCT, single-blind	150	*Cocos nucifera*, acetic acid	Coconut oil and vinegar or coconut oil	2 treatments for 8hr (D1, D8)	CO 32/50 (64%) CV 47/50 (94%)	None	Permethrin 1% shampoo	2 treatment for 5 min (D1, D8)	49/50 (98%, p = 0.00)	Eye irritation: 8 Irritation^a^: 3
Burgess et al. (2009)	RCT, single-blind	100	*Cocos nucifera*, *Pimpinella anisum*	Fractionated coconut oil, propan-1-ol, anise oil, and ylang-ylang oil spray	2 treatments for 15min (D1, D9)	46/50 (92.0%)	Irritation^a^: 17	Permethrin 0.43% spray	2 treatments for 45 minutes (D1, D9)	24/48 (50%, p < 0.0001)	Irritation^a^: 20
Scanni (2005)	RCT, non-blinded	24	*Cananga odorata*, *Cocos nucifera*, *Pimpinella anisum*	Coconut oil, anise and ylang ylang oil spray (Paranix)	3 treatments for 15min (D0, D6, D11)	11/11 (100%)	Odour: 1	Malathion 0.5%	2 treatments for 10min (D0, D7)	11/11 (100%, p = NR)	Odour: 2
Soonwera (2014)^b^	RCT, single-blind	210	*Acorus calamus* Linn., *Phyllanthus emblica* Linn., *Zanthoxylum limonella* Alston	10% w/v crude extract of *Acorus calamus* rhizomes., *Phyllanthus emblica*. fruits, *Zanthoxylum limonella* fruits	2 treatments for 15min (D1, D7)	Absolute rate NR (100%)	None	Malathion shampoo 1% shampoo Carbaryl 0.6% shampoo Babi Mild Natural N Mild shampoo Johnson’s baby shampoo	2 treatments for 15 min (D1, D7)	Absolute rate NR Malathion shampoo (85.33%, p < 0.05) Carbaryl shampoo 93%, Babi Mild Natural N Mild shampoo (0%, p < 0.05) Johnson‘s Baby shampoo (0%, p < 0.05)	Irritation^a^: Present but NR no. events
Barker & Altman (2010)	RCT, single-blind	123	*Melaleuca alterniflora*, *Lavandula*	Tea tree oil 10% and lavender oil 1% (NeutraLice Lotion)	3 treatments for 10min (D0, D7, D14)	41/42 (97.6%)	Irritation^a^: 29	Pyrethrin 1.65mg/g/ piperonyl butoxide 16.5mg/g (BanLice Mousse)	2 treatments for 10 minutes (D0, D7)	10/40 (25.0%, p < 0.0001)	Irritation^a^: 4
Tiangda (2000)^b^	RCT, non-blinded	22	*Annosa squamosa* Linn.	Custard apple seed extract 20% w/w oil in water cream	1 treatment for 3hr	Proportion of dead lice: 261/274 (95.3%)	None	Control (cream base) Benzyl benzoate 25% emulsion with fine combing	1 treatment for 3hr	Proportion of dead lice: Cream base 31/73 (47.4%, p < 0.05), Benzyl benzoate 15/33 (60.1%, p < 0.05)	Irritation^a^: Present but NR no. events

NR- not reported

a: Irritation: itch, stinging and/or burning

b. Not included in quantitative analysis as no cure rates reported in studies.

c: Groups do not compare neem vs no neem but rather combing vs combing with placebo comb

**Table 2:** Summary of non-randomized controlled trials.

Author (Year)	N=	Plant/s (Scientific)	Intervention	Dose, duration, frequency	Intervention final cure rate (%)	Comparator	Adverse events
Al Zayadi (2020)	45	*Lawsonia inermis*, C*urcuma longa*, *Allium cepa*	Red onion 150g and ground turmeric 10g; or Vinegar 250ml and salt 25g	1 treatment for 2 hrs	Onion juice and turmeric 12/15 (80%) Vinegar and salt 7/15 (46.7%)	None	NR
Thawornchaisit et al., (2012)	45	*Azadirachta indica*, *Eucalyptus* spp.	Neem oil 6% and eucalyptus oil 16% in carrier lanolin+ silicone	2 treatments for 30min (D0, D7)	Absolute rate NR (89%)	None	Allergic contact dermatitis: 1
Abdel-Ghaffar et al., (2012) single arm trial	20	*Azadirachta indica*	Neem shampoo (Licener)	1 treatment for 10 or 20 min	20/20 (100%)	None	NR
Abdel-Ghaffar and Semmler (2006)	60	*Azadirachta indica*	Neem with variable duration (3 arms) *	1 -2 treatments for 5–30min (D1, D10) *	Absolute rate NR (86 – 97%)	None	Irritation*
Abdel-Ghaffar et al., (2009)	20	*Citrus × paradisi*	Grapefruit (Licatack)	1 treatment for 10 min or 20 min	18/18 (100%)	Pure tap water (N = 2) 0%	None
McCage et al., (2002)	16	*Asimia triloba Dunal, Thymus vulgaris, Melaleuca alternifolia* (Maiden and Betche) Cheel	TTO 0.5%, Thymol 1%, Pawpaw extract 0.5%, Shampoo 98%	3 treatments for 1hr (D0, D7, D14)	16/16 (100%)	None	None
El-Bashier and Fouad, (2002)	100	*Lawsonia alba* L., *Trigonella-faemum-gracanum*, *Hibiscus cannabinus*, *Artemisia cina*	Henna, helba; or Henna, karkade; or Henna, sheah	3 treatments for 3hrs (between D1 – D7)	Henna, helba-75/100 (75%) Henna, karkarde 50/100 (50%) Henna, sheah 100/100 (100%)	None	NR

All studies are single-arm trials with exception of Abdel and Ghaffar et al., (2009)

All studies are open label

*Methodology or number of participants involved not clearly defined for treatment duration, dosing, frequency, or adverse events

### Characteristics of included studies

Five studies were conducted in each of Egypt and Asia, 3 studies in Australia and the Middle East, 2 studies in the UK and one study in Brazil and the USA. Almost half of the studies (9 studies, 45%) had sample size less than 50. Four studies (20%) had 50–99 participants, 6 studies (30%) had participants of 100–149 and only 2 studies (10%) had participants over 150. Eighteen (90%) studies included both females and males, 3 (15%) studies included only females, and 2 (10%) studies did not include gender. There was a total of 1575 participants included in the RCTs and NRCTs. Fifteen (75%) studies included only children, 3 (15%) studies included children and adults, and 2 (10%) studies did not record age [S1 Table].

### Treatment characteristics

All treatments were topical. The most common interventions were neem (5 studies, 25%), anise (3 studies, 15%) and eucalyptus oil (2 studies, 10%). Most studies had 2 treatments applied every 7–9 days (6 studies, 30%), followed by 3 treatments every 5–10 days (7 studies, 30%) and single treatment (5 studies, 22%). One study (4%) had 6 treatments, with 3 initial treatments repeated after 1 week.

### Mechanism of action

No studies directly investigated or reported the mechanism of action of plant phytochemicals. Quoted mechanisms include suffocation, microtubule formation and ATP depletion but these hypotheses were not supported in the studies. Cardoso (2020) suggest that Eur-Amid N2 and phytosterols help dissolve epicuticular waxes covering the parasite cuticles. The resin then hardens, causing parasite cuticle cracking, resulting in biological function and external defence breakdown [36].

### Detection method

Twelve (60%) studies utilized a nit comb only as the detection method, 4 (20%) studies used visual examination and 3 (15%) studies used wet combing. One study (5%) did not document the detection method. Ten (50%) studies documented final cure rates, and 4 (20%) studies documented initial cure rates. There are no data that compares the effect of detection method on outcomes in any of the included studies.

### Cure rates

#### Randomized clinical trials.

Thirteen RCTs were included in qualitative analysis, with 4 excluded from quantitative analysis: 3 due to cure rates not being reported [39–41] and one because the concentration of 0.43% permethrin was below the commonly used concentration of 1% [32]. Eleven studies were included in the quantitative analysis, with 11 sets of data, noting 2 studies had 2 intervention arms. [Table 3, S2 Table]

**Table 3:** Study cure rates within categories: 14-day follow-ups (n = 11).

Study	Treatment	Plant-based	Conventional treatment	Rate ratio
Anise based				
Mumcuoglu 2002	CO/A/Y vs P/PB	0.86	0.81	1.06
Scanni 2005	CO/A/Y vs P/PB	1.00	1.00	1.00
Eucalyptus oil based				
Greive and Altman 2007	EO/LP vs P/PB	0.83	0.36	2.28
Greive and Barnes 2017	EO/LP vs P/PB	0.83	0.36	2.28
Greive and Altman 2007	EO/LP vs malathion	0.83	0.3	2.77
Other				
Barker & Altman 2010	TTO/ Lavender vs P/PB	0.98	0.25	3.90
Semmler 2017	Neem vs Dimethicone	1.00	0.96	1.04
Maarefvand 2019	P. harmala vs P	0.92	0.87	1.06
Cardoso 2020	P. hepatophyllum vs P	0.95	0.39	2.44
Moreno-Alsasua 2016	CO vs P	0.64	0.98	0.65
Moreno-Alsasua 2016	CO/V vs. P	0.64	0.98	0.65

Abbreviations:

CO/A/Y: Coconut Oil/Anise/Ylang Ylang

P/PB: Pyrethrin/Piperonyl Butoxide

TTO: Tea tree oil

P: Permethrin

V: Vinegar

The overall average final cure rate at 14-day follow-up for materials of plant origin was 0.86 (95% CI 0.73, 0.99), which was significantly higher than conventional treatment at 0.60 (95% CI 0.25, 0.95) with a mean difference of 0.26 (95% CI 0.18, 0.35, p < 0.001). Significant differences in mean cure rate were observed for overall plant-based treatments and EO-based treatments when compared to conventional treatment (p < 0.001), but not Anise treatments (p = 0.789) [Table 4].

**Table 4:** Average measures: 14-day follow-ups (n = 11).

		Plant-based	Conventional treatment	Difference	
Category	n	Mean (SD)	Mean (SD)	Mean (95%CI)	P^#^
All	11	0.86 (0.13)	0.60 (0.35)	0.26 (0.18, 0.35)	<0.001
Anise	2	0.93 (0.10)	0.71 (0.40)	0.03 (-0.88, 0.93)	0.789
EO	3	0.83 (0.00)	0.34 (0.04)	0.49 (0.48, 0.49)	<0.001
Other	6	0.86 (0.17)	0.62 (0.35)	0.12 (-0.06, 0.29)	0.154

# difference between treatment and control

Abbreviations:

EO: Eucalyptus oil

Other: Tea tree oil, lavender, neem, *P. harmala*, *P. hepatophyllum*

For the 4 studies that performed repeated follow ups there was a significant increase in mean rate of cures in the combined plant-based arms from day 7 to day 14 (p = 0.005) whereas there was no change in mean cure rate in the combined insecticide arms (p = 0.338), suggesting that the extra time improved plant-based cure rates. The rate ratio (PB/I) remained constant for both follow ups. [Table 5]

**Table 5:** Repeated measures: 7- and 14-day follow-ups (n = 4).

Treatment	Day 7	Day 14	Difference 7–14 days	P*
	Mean (95%CI)	Mean (95%CI)	Mean (95%CI)	
Plant-based	0.68 (0.53, 0.82)	0.92 (0.78, 1.07)	0.25 (0.08, 0.42)	0.005
Conventional	0.49 (0.24, 0.75)	0.66 (0.40, 0.91)	0.16 (-0.17, 0.50)	0.338
Rate ratio	1.67 (1.01, 2.33)	1.70 (1.04, 2.35)	0.03 (-0.90, 0.96)	0.952

* difference over time (day 7 to day 14)

N = 11 studies were entered into a random effects meta-analysis with n = 1 excluded by the analysis^38^. Overall effect favoured plant-based interventions compared to conventional treatment (OR 3.79, 95% CI 1.23, 11.66), with high heterogeneity of studies (I^2^ = 83.2%).

**Fig 2:**
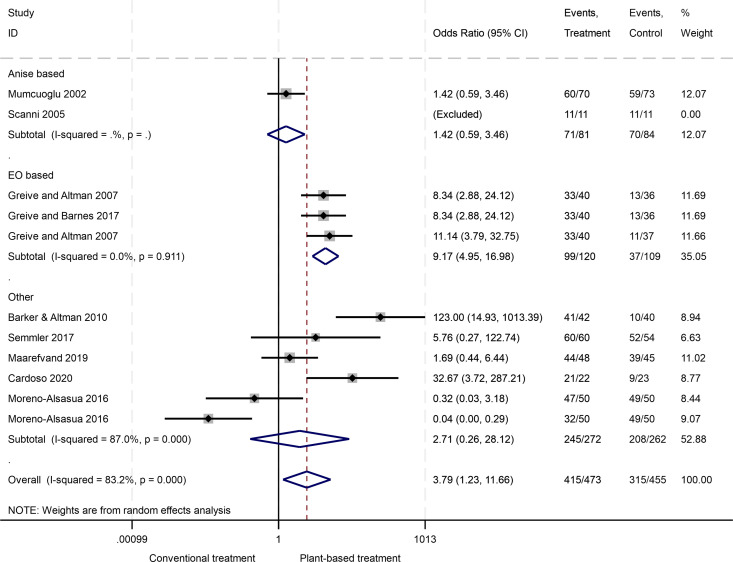
Meta-analysis of 14-day studies based on treatment categories comparing odds of cure with conventional treatment vs. plant-based treatment.

EO based treatments were associated with significantly higher odds of cure compared to insecticides (OR 9.17, 95% CI 4.95, 16.98), I^2^ = 0%. Other treatments had higher odds of cure than insecticides but was not statistically significant (OR 2.71, 95% CI 0.26, 28.12), I^2^ = 87.0%. Anise based treatments showed higher odds of cure compared to insecticides but was not statistically significant (OR 1.42, 95% CI 0.59, 3.46). Other categories had high study heterogeneity (I^2^ = 86.5% and 80.9%, respectively). [Fig 2].

### Non-randomized clinical trials

Seven NRCTs were analysed qualitatively. All studies were single arm trials, with no control groups. The application of henna and sheah resulted in 75% cure rate [43]. A single arm trial of 20 participants exposed to Grapefruit extract reported all lice remained motionless after treatment of 10 or 20 minutes [46]. The use of neem oil 6% and EO 16% in hydrophobic carrier demonstrated absence of detection of live lice in 42/44 participants (96%) on day 3 but this reduced to 39/44 (89%) on day 14 likely due to reinfestation [47]. [Table 2, S3 Table].

### Adverse events

Adverse events were documented in 20 studies (87%), with no systemic or severe events. Neem reported low adverse events in 83% of studies, however one episode of allergic contact dermatitis was reported with application of neem 6% and EO 16% [49]. Anise-based products were also well tolerated, with only mild irritation (13%) and odour (2.4%) reported [30,32]. EO 11% with *Leptospermum petersonii* 1% (MOOV Headlice Solution) was associated with transient itching, burning and stinging in 45% of participants [31,42]. In a study with TTO/ lavender oil, 30.2% experienced stinging, 18.6% had flaky/ dry scalp and 9.3% had erythema [33]. No adverse events were experienced from *Protium hepatophyllum*, grapefruit extract, henna paste, onion, curcumin and custard apple [34,40,46,48]. However, adverse event reporting was not standardized across studies.

### Risk of bias

**Fig 3:**
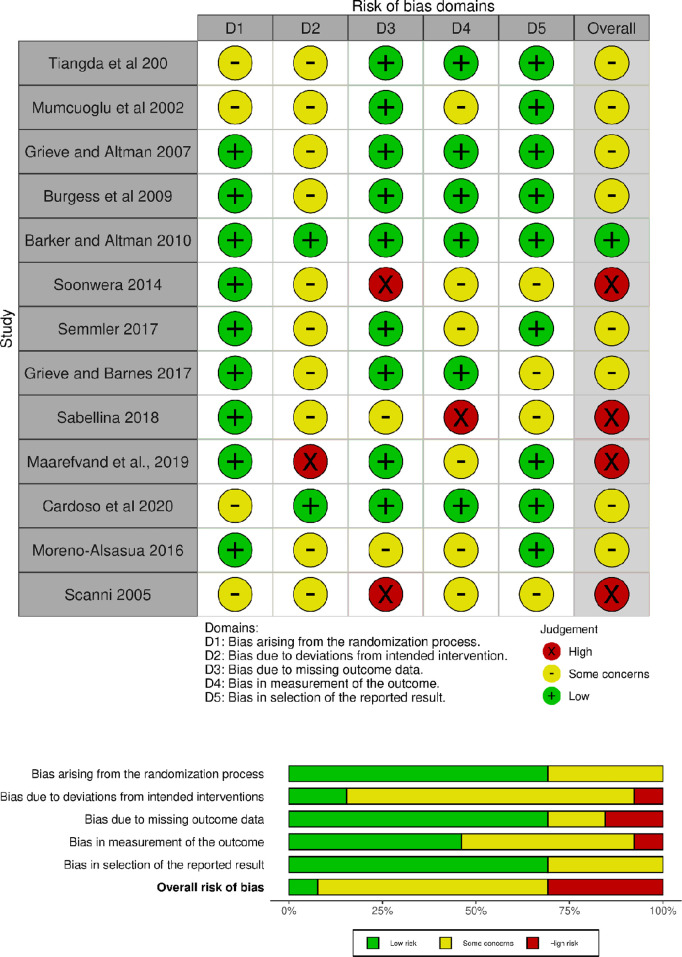
Risk of bias RCT.

The overall risk of bias for RCTs were of some concerns (8 studies, 62%) or high concerns (4 studies, 31%). The domains of greatest concern in the RCTs were bias due to deviations from intended interventions due to the lack of blinding. There was measurement bias in 1 study, where the outcome was measured after combing the hair 6 times [Fig 3] [34].

**Fig 4:**
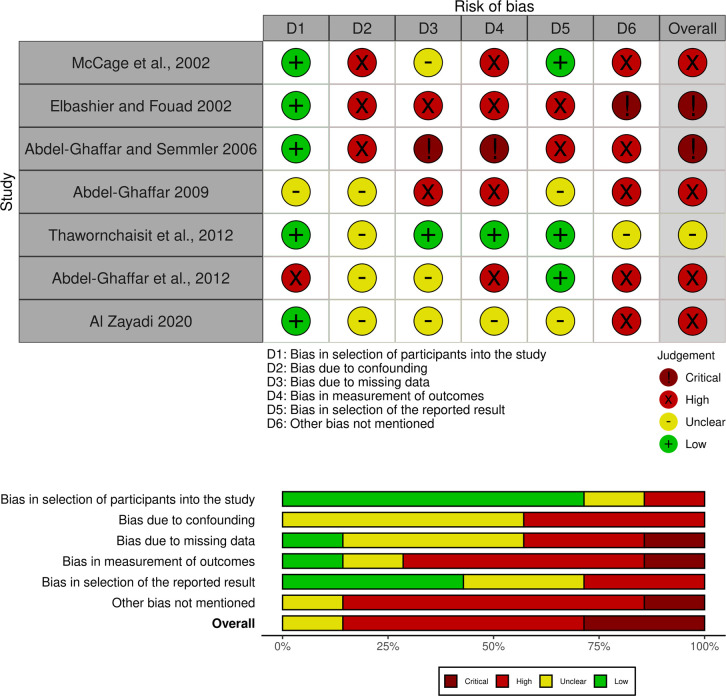
Risk of Bias NRCT.

The overall risk of bias for NRCTs were demonstrated to be high (3 studies, 43%), followed by critical and moderate in 1 studies (29%) respectively. The domains of greatest concern were bias due to missing data and outcome measurements. Most of the studies were composed of small participant numbers. Baseline characteristics were poorly defined in several studies [43,48]. There was limited information trial and detection methods [43,45]. [Fig 4]

**Fig 5:**
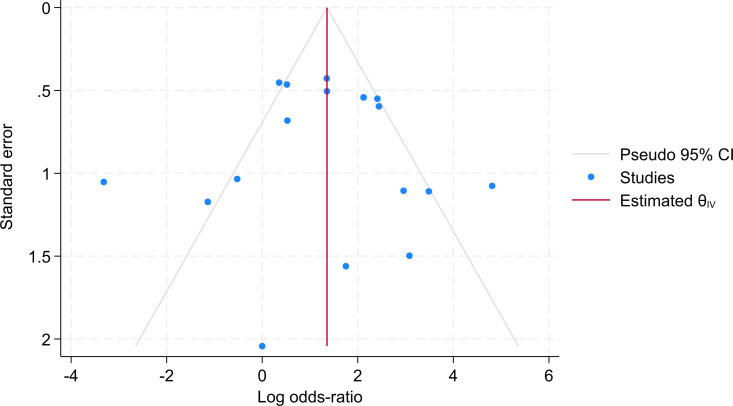
Funnel plot of studies.

A funnel plot revealed a reasonably symmetric shape, indicating an absence of publication bias (Fig 5).

## Discussion

This is the first systematic review and meta-analyses evaluating clinical efficacy of plant-based therapies in treating PC. Based on data from RCTs, this review suggests that certain plant-based interventions may offer improved cure rates compared to conventional treatments, but these findings are limited by study heterogeneity and methodological constraints.

One might not be able to conclude all plants are effective towards PC, as plants analysed in this review were a small diverse group of individual plants. Based on subgroup analysis, only some plant-based treatments demonstrated promising results such as Eucalyptus oil*/ Leptospermum petersonii*, TTO/ lavender, and *Protium hepatophyllum*, although these findings stem from a limited number of trials, and further high quality RCTs are needed to confirm their efficacy. Our study demonstrates an 86% (95% CI 0.73, 0.99) cure rate for plant-based intervention which is higher than the conventional treatment cure rate of 60% (95% CI 0.25, 0.95). Our average cure rate for conventional treatment was comparable to efficacy rates from other meta-analysis (59%, 61%) [50,51]. To date, few guidelines include plant treatments, possibly due to expense, supply and evidence. Significant outcomes were achieved with earlier-time interval analysis suggesting effectiveness after single treatment. Several plant materials evaluated in NRCTs are not supported by RCT data, such as grapefruit, henna, turmeric and red onion. Due to their lack methodological rigor, NRCTs alone are insufficient to conclude their clinical effectiveness despite high cure rates.

Adverse events reported within studies were limited to local irritation without systemic adverse events. However, adverse events depend on the plant material used which is not consistent between studies. Additionally standardized methods of adverse event reporting were not utilised across studies. This is reflected by the frequency of adverse events being highly variable, ranging from minimal to no participants to nearly half of participants using eucalyptus oil experiencing transient mild to moderate scalp itching, burning and stinging [42]. However, the study comment that “the treatments were well tolerated” does not directly evaluate whether this had an effect on treatment adherence [42]. Although the lack of systemic symptoms suggests a relatively safe therapeutic option, adverse events are not well described or recorded among included studies therefore should be cautiously interpreted.

Neem carries longstanding historical significance in Ayurvedic medicine as an anti-microbial agent and was investigated in two RCTs and three single arm trials [34,41,44,45,47,48]. Over 100 biologically active ingredients exist with major constituents including limonoids and nimbin [52]. However, the effectiveness of neem in our review was unable to be supported due to small study size, study heterogeneity in study design, and poorer quality NRCTs. Neem was not more effective than permethrin in reducing head lice count in one RCT [41]. We also note another RCT by Brown and Burgess (2017) evaluating a 1% neem-based lotion with a grooming comb against a nit comb which found no significant difference in combing method, with both arms being ineffective in treatment of PC [53].

We offer several alternative reasons regarding our observed superiority of plant-based interventions. A possible explanation includes the large resistance rates previously documented with permethrin [18]. These studies are conducted in different geographical areas with varying resistance rates to conventional insecticides which may impact results. This widespread resistance may have been acknowledged by investigators and selected as comparators to enhance the plant-based intervention outcomes. However, resistance testing was not acknowledged or systematically conducted in the included studies, therefore this is only hypothesized. Plant-based interventions may be less likely to develop resistance, due to their volatility and multimodal mechanism of actions [32,37]. One comparator used a lower dose of permethrin of 0.43% compared to the most common permethrin preparation of 1% which may introduce bias [32]. For this reason, this study was excluded from our analyses.

This review does not directly assess the in vitro efficacy of plant-based interventions, however previous reviews have well described the effectiveness of numerous plants [54,55]. Some of our studies also had an in vitro component [36,39,44,47]. One large in vitro study examined the activity of 54 essential oils against pyrethrin and found sixteen with equal or greater efficacy [56]. Candy et al summarised 172 studies of essential oils against PC, describing 22 major plant genera, and their major active constituents including abundant mono-oxygenated compounds, phenylpropanoids and monoterpene hydrocarbons [55]. Some essential oils were effective against permethrin-resistant PC, as well as dual resistant pyrethroid/ malathion resistant lice [57,58]. Many plant species such as *Cinnamomum* with the highest effectiveness in in vitro testing have not been evaluated in clinical trials [56]. However, similar to clinical trials, in vitro studies lack protocol standardisation such as variable assay utilisation (filter paper, immersion or fumigation assays), mortality definitions, head lice sources, and use of controls [55,56]. Despite the substantial in vitro evidence base to support the insecticidal activity of plants, there is a relative lack of correlative in vivo testing. In vitro studies require further validation through standardized clinical trials to prove their clinical effectiveness.

The precise mechanism of action of plant-based therapies has not been fully evaluated. Several manufacturers supplying products used in clinical trials claim suffocation as the primary mechanism such as for coconut oil, neem, tea tree oil/ lavender oil and grapefruit. However, this hypothesis is not directly investigated or supported in the study itself. Evidence suggests the mechanism of action of plants is multi-modal. For example, essential oils such as tea tree oil typically have neurotoxic activity, through monoterpenoid mediated acetylcholine esterase inhibition similar to organophosphate insecticides [58]. The effect of the plant may also be confounded by other ingredients. For example, neem formulations used in several studies [34,43,46–48] contain powerful emulsifying shampoos which may exert dehydrating effects on lice [59].

### Limitations

The limitations of our systematic review include small sample sizes, high heterogeneity in methodology (dosage, frequency, application techniques) and lack of blinding. Wet combing was only performed in three of the studies despite being the standard method of detection. Generalizability of results may be limited due to variable concentration of active ingredients and availability of native plant material. Seasonal availability, geography and soil may also contribute to different plant concentrations. Several studies were funded by pharmaceuticals companies [32,33,39,42] which may introduce bias. We only included English language studies although PC is experienced globally.

The risk of bias for NRCTs were high or critical suggests that these studies do not provide sufficient evidence for their clinical effectiveness. RCTs were demonstrated to have some concerns.

Multiple confounding factors were identified. For example, in one study, the male participants’ hair was cut short to remove PC, and in another the participant’s hair was combed six times prior to assessment [34,49]. Across studies there was significant variability in reported treatment schedules (e.g., single vs. multiple applications), with the justification often not specified or based on manufacturer instructions. Although gender was extracted in our study, correlative factors such as hair length were unable to be analyzed due to study data insufficiency and heterogeneity. Some studies performed in community settings may affect transmission and reinfestation. This is reflected in one study where the rate of recurrence on the 14^th^ day was higher compared to 9^th^ day, attributed to potential reinfestation [47]. Multiple active ingredients in combination therapies may also have synergistic or antagonistic effects. Plant compounds also have complex phytochemical compositions not formally analysed or reported in studies. The exact contribution of each phytochemical or carrier are not differentiated within studies. Additionally, studies rarely report the abundance of lice found at each stage of development and time point, which may signify whether persistent lice are insensitive to treatment or newly acquired. For example, subadults found days after treatment may have been protected embryos within the eggs during treatment.

## Conclusion

Results should be cautiously interpreted in context of high study heterogeneity and moderate to high risk of bias. Further clinical studies should incorporate standardized comparators, detection methods, standardized adverse event reporting, treatment protocols and outcomes. Ideally, larger RCTs will provide higher quality evidence, with consideration of other therapeutic plants identified from in vitro studies with rigorous scientific foundation including investigation of mechanism of action. To improve compliance, further data on shorter cure rate periods (i.e., 24-hours post application) would be beneficial in identifying rapidly efficacious plants. Validated questionnaires assessing patient satisfaction, quality of life and ease of use ought to be considered. Future studies should also consider health economic analysis, availability of products, quality control and sub-analyses of individual phytochemicals for their efficacy. Limitations to inclusion of plant derived materials and others of natural origin in current guidelines may be attributed to lack of current evidence, small study sizes, lack of knowledge of mechanism of action, efficacy and adverse events. Further research is required to elucidate the role of plant-based therapies in PC in clinical practice. This study encourages the ongoing research and development of plant-based therapies, which is essential in the face of emerging resistance to conventional therapies.

## Supporting information

S1 Table Characteristics of included studies. (DOCX)

S2 Table Summary of randomized controlled trials. (DOCX)

S3 Table Summary of non-randomized controlled trials. (DOCX)

S1 File Search strategy with full list of included studies. (XLSX)

S2 File PROSPERO protocol with post peer review amendments. (DOCX)

## References

[1] Stevenson B, Tesfaye W, Christenson J, Mathew C, Abrha S, Peterson G, et al. Comparative efficacy and safety of interventions for treating head lice: a protocol for systematic review and network meta-analysis. BMJ Paediatr Open. 2021;5(1):e001129. doi: 10.1136/bmjpo-2021-001129 34041368 PMC8112437

[2] Thomas HM, Enkel S, McRae T, Cox V, Kessaris HL, Ford AJ. Skin health in northern Australia. Microbiol Aust. 2022;43(3):98–103.

[3] Counahan M, Andrews R, Büttner P, Byrnes G, Speare R. Head lice prevalence in primary schools in Victoria, Australia. J Paediatr Child Health. 2004;40(11):616–9. doi: 10.1111/j.1440-1754.2004.00486.x 15469530

[4] Moosazadeh M, Afshari M, Keianian H, Nezammahalleh A, Enayati AA. Prevalence of Head Lice Infestation and Its Associated Factors among Primary School Students in Iran: A Systematic Review and Meta-analysis. Osong Public Health Res Perspect. 2015;6(6):346–56. doi: 10.1016/j.phrp.2015.10.011 26835244 PMC4700766

[5] Bachok N, Nordin RB, Awang CW, Ibrahim NA, Naing L. Prevalence and associated factors of head lice infestation among primary schoolchildren in Kelantan, Malaysia. Southeast Asian J Trop Med Public Health. 2006;37(3):536–43. 17120976

[6] Currie MJ, Reynolds GJ, Glasgow NJ, Bowden FJ. A pilot study of the use of oral ivermectin to treat head lice in primary school students in Australia. Pediatr Dermatol. 2010;27(6):595–9. doi: 10.1111/j.1525-1470.2010.01317.x 21138467

[7] Oh J-M, Lee IY, Lee W-J, Seo M, Park S-A, Lee SH, et al. Prevalence of pediculosis capitis among Korean children. Parasitol Res. 2010;107(6):1415–9. doi: 10.1007/s00436-010-2016-6 20683614

[8] Mahmud S, Pappas G, Hadden WC. Prevalence of head lice and hygiene practices among women over twelve years of age in Sindh, Balochistan, and North West Frontier Province: National Health Survey of Pakistan, 1990-1994. Parasit Vectors. 2011;4:11. doi: 10.1186/1756-3305-4-11 21288357 PMC3040706

[9] Buczek A, Markowska-Gosik D, Widomska D, Kawa IM. Pediculosis capitis among schoolchildren in urban and rural areas of eastern Poland. Eur J Epidemiol. 2004;19(5):491–5. doi: 10.1023/b:ejep.0000027347.76908.61 15233324

[10] Ricciardo BM, Kessaris H-L, Nannup N, Tilbrook D, Farrant B, Michie C, et al. Describing skin health and disease in urban-living Aboriginal children: co-design, development and feasibility testing of the Koolungar Moorditj Healthy Skin pilot project. Pilot Feasibility Stud. 2024;10(1):6. doi: 10.1186/s40814-023-01428-6 38200545 PMC10782716

[11] Custodio J, Kelly G, Haenga M, Bell C, Bond T, Prouse I. Working in partnership with communities at risk: the potential of integrated public health action during an outbreak of APSGN in remote Australia. Australian Indigenous Health Bulletin. 2016;16(4):1–12.

[12] Guss DA, Koenig M, Castillo EM. Severe iron deficiency anemia and lice infestation. J Emerg Med. 2011;41(4):362–5. doi: 10.1016/j.jemermed.2010.05.030 20656443

[13] Althomali SA, Alzubaidi LM, Alkhaldi DM. Severe iron deficiency anaemia associated with heavy lice infestation in a young woman. BMJ Case Rep. 2015;2015:bcr2015212207. doi: 10.1136/bcr-2015-212207 26542960 PMC4654199

[14] Roberts RJ, Casey D, Morgan DA, Petrovic M. Comparison of wet combing with malathion for treatment of head lice in the UK: a pragmatic randomised controlled trial. Lancet. 2000;356(9229):540–4. doi: 10.1016/s0140-6736(00)02578-2 10950230

[15] Leung AKC, Lam JM, Leong KF, Barankin B, Hon KL. Paediatrics: how to manage pediculosis capitis. Drugs Context. 2022;11:2021-11–3. doi: 10.7573/dic.2021-11-3 35371269 PMC8932250

[16] Verma P, Namdeo C. Treatment of Pediculosis Capitis. Indian J Dermatol. 2015;60(3):238–47. doi: 10.4103/0019-5154.156339 26120148 PMC4458933

[17] Stough D, Shellabarger S, Quiring J, Gabrielsen AA Jr. Efficacy and safety of spinosad and permethrin creme rinses for pediculosis capitis (head lice). Pediatrics. 2009;124(3):e389-95. doi: 10.1542/peds.2008-3762 19706558

[18] Nolt D, Moore S, Yan AC, Melnick L. Committee on Infectious Diseases; Section on Dermatology. Head lice. Pediatrics. 2022;150(4):e2022059282. 10.1542/peds.2022-059282 36156158

[19] Mohammadi J, Azizi K, Alipour H, Kalantari M, Bagheri M, Shahriari-Namadi M. Frequency of pyrethroid resistance in human head louse treatment: systematic review and meta-analysis. Parasite. 2021;28. 10.1051/parasite/2021083 PMC8693761 34935614

[20] Durand R, Bouvresse S, Berdjane Z, Izri A, Chosidow O, Clark JM. Insecticide resistance in head lice: clinical, parasitological and genetic aspects. Clin Microbiol Infect. 2012;18(4):338–44. doi: 10.1111/j.1469-0691.2012.03806.x 22429458

[21] Pariser DM, Meinking TL, Bell M, Ryan WG. Topical 0.5% ivermectin lotion for treatment of head lice. N Engl J Med. 2012;367(18):1687–93. doi: 10.1056/NEJMoa1200107 23113480

[22] Ameen M, Arenas R, Villanueva-Reyes J, Ruiz-Esmenjaud J, Millar D, Domínguez-Dueñas F. Oral ivermectin for treatment of pediculosis capitis. J Pediatr Infect Dis. 2010;29(11):991–3. 21046698

[23] Chosidow O, Giraudeau B, Cottrell J, Izri A, Hofmann R, Mann SG, et al. Oral ivermectin versus malathion lotion for difficult-to-treat head lice. N Engl J Med. 2010;362(10):896–905. doi: 10.1056/NEJMoa0905471 20220184

[24] Deeks LS, Naunton M, Currie MJ, Bowden FJ. Topical ivermectin 0.5% lotion for treatment of head lice. Ann Pharmacother. 2013;47(9):1161–7. doi: 10.1177/1060028013500645 24259731

[25] Amanzougaghene N, Fenollar F, Diatta G, Sokhna C, Raoult D, Mediannikov O. Mutations in GluCl associated with field ivermectin-resistant head lice from Senegal. Int J Antimicrob Agents. 2018;52(5):593–8. doi: 10.1016/j.ijantimicag.2018.07.005 30055248

[26] Absil G, Lebas E, Libon F, El Hayderi L, Dezfoulian B, Nikkels AF. Scabies and therapeutic resistance: Current knowledge and future perspectives. JEACP. 2022;1(3):157–64.

[27] Bomze D, Sprecher E, Geller S. Severe cutaneous adverse reactions associated with systemic ivermectin: A pharmacovigilance analysis. J Dermatol. 2022;49(8):769–74. doi: 10.1111/1346-8138.16398 35475524 PMC9111106

[28] Sterne JA, Savović J, Page MJ, Elbers RG, Blencowe NS, Boutron I, et al. RoB 2: a revised tool for assessing risk of bias in randomised trials. BMJ. 2019;366. 10.1136/bmj.l4898 31462531

[29] Sterne JA, Hernán MA, Reeves BC, Savović J, Berkman ND, Viswanathan M. ROBINS-I: a tool for assessing risk of bias in non-randomised studies of interventions. BMJ. 2016;355. 10.1136/bmj.i4919 PMC5062054 27733354

[30] Mumcuoglu KY, Miller J, Zamir C, Zentner G, Helbin V, Ingber A. The in vivo pediculicidal efficacy of a natural remedy. Isr Med Assoc J. 2002;4(10):790–3. 12389342

[31] Greive KA, Barnes TM. The efficacy of Australian essential oils for the treatment of head lice infestation in children: A randomised controlled trial. Australas J Dermatol. 2018;59(2):e99–105. doi: 10.1111/ajd.12626 28266704 PMC6001441

[32] Burgess IF, Brunton ER, Burgess NA. Clinical trial showing superiority of a coconut and anise spray over permethrin 0.43% lotion for head louse infestation, ISRCTN96469780. Eur J Paediatr. 2010;169(1):55–62. 10.1007/s00431-009-0978-0 19343362

[33] Barker SC, Altman PM. A randomised, assessor blind, parallel group comparative efficacy trial of three products for the treatment of head lice in children--melaleuca oil and lavender oil, pyrethrins and piperonyl butoxide, and a “suffocation” product. BMC Dermatol. 2010;10:6. doi: 10.1186/1471-5945-10-6 20727129 PMC2933647

[34] Semmler M, Abdel-Ghaffar F, Gestmann F, Abdel-Aty M, Rizk I, Al-Quraishy S, et al. Randomized, investigator-blinded, controlled clinical study with lice shampoo (Licener®) versus dimethicone (Jacutin® Pedicul Fluid) for the treatment of infestations with head lice. Parasitol Res. 2017;116(7):1863–70. doi: 10.1007/s00436-017-5461-7 28488042

[35] Maarefvand M, Kenari HM, Ghobadi A, Soleymani A, Hashem-Dabaghian FHD. Efficacy of the Peganum harmala oil versus 1% permethrin shampoo on the treatment of head louse infestation. J Pharm Res Int. 2019;27(6):1–9.

[36] Leal Cardoso JH, Noronha Coelho de Souza A, Militão de Souza F, Sa Preire S, Pinçon C. Treatment of head louse infestation with a novel mixture made of semi-crystalline polymers and plant extracts: blind, randomized, controlled, superiority trial. Cosmetics. 2020;7(2):25.

[37] Moreno-Alsalsua M. Randomized controlled trial on the effect of coconut oil, vinegar plus cooking coconut oil versus 1% permethrin shampoo in the treatment of pediculosis. Ped Infect Dis Soc Phil J. 2016;17(2):4–13.

[38] Scanni G, Bonifazi E. Efficacy of a single application of a new natural lice removal product. Preliminary data. Eur J Pediatr. 2006;16(4):231.

[39] Soonwera M. Efficacy of herbal shampoo base on native plant against head lice (Pediculus humanus capitis De Geer, Pediculidae: Phthiraptera) in vitro and in vivo in Thailand. Parasitol Res. 2014;113(9):3241–50. doi: 10.1007/s00436-014-3986-6 24948104

[40] Tiangda CH, Gritsanapan W, Sookvanichsilp N, Limchalearn A. Anti-headlice activity of a preparation of Annona squamosa seed extract. Southeast Asian J Trop Med Public Health. 2000;31 Suppl 1:174–7. 11414452

[41] Sabellina LAN, Salamanca CSS, Santos DEA, Seron MA, Planes AB, Policarpio MA, et al. Effectiveness of neem seed oil methanolic extract shampoo versus permethrin shampoo in the reduction of head lice infestation in children. UERM Health Sci J. 2018;58.

[42] Grieve K, Altman P, Rowe S, Staton J, Oppenheim V. A randomised, double-blind, comparative efficacy trial of three head lice treatment options: malathion, pyrethrins with piperonyl butoxide and MOOV Head Lice Solution. Aust Pharmacist. 2007;26(9):738–43.

[43] El-Basheir ZM, Fouad MAH. A preliminary pilot survey on head lice, pediculosis in Sharkia Governorate and treatment of lice with natural plant extracts. J Egypt Soc Parasitol. 2002;32(3):725–36. 12512805

[44] Abdel-Ghaffar F, Al-Quraishy S, Al-Rasheid KAS, Mehlhorn H. Efficacy of a single treatment of head lice with a neem seed extract: an in vivo and in vitro study on nits and motile stages. Parasitol Res. 2012;110(1):277–80. doi: 10.1007/s00436-011-2484-3 21667206

[45] Abdel-Ghaffar F, Semmler M. Efficacy of neem seed extract shampoo on head lice of naturally infected humans in Egypt. Parasitol Res. 2007;100(2):329–32. doi: 10.1007/s00436-006-0264-2 16900389

[46] Abdel-Ghaffar F, Semmler M, Al-Rasheid K, Klimpel S, Mehlhorn H. Efficacy of a grapefruit extract on head lice: a clinical trial. Parasitol Res. 2010;106:445–9. 19943066 10.1007/s00436-009-1683-7

[47] Thawornchaisit P, Amornsak W, Mahannop P, Buddhirakkul P, Pandii W, Connellan P, et al. Combined neem oil 6% w/w and eucalyptus oil 16% w/w lotion for treating head lice: in vitro and in vivo efficacy studies. J Pharm Pract Res. 2012;42(3):189–92.

[48] Sundus Wafi Al-Zayyadi. Study of the Effectiveness of Some Raw Plants and Materials in the Treatment of Pediculosis in Najaf province Iraq. Indian Journal of Forensic Medicine & Toxicology. 2020;14(1):499–503. doi: 10.37506/ijfmt.v14i1.96

[49] McCage CM, Ward SM, Paling CA, Fisher DA, Flynn PJ, McLaughlin JL. Development of a paw paw herbal shampoo for the removal of head lice. Phytomedicine. 2002;9(8):743–8. doi: 10.1078/094471102321621377 12587697

[50] Flores-Genuino RNS, Gnilo CMS, Dofitas BL. Occlusive versus neurotoxic agents for topical treatment of head lice infestation: A systematic review and meta-analysis. Pediatr Dermatol. 2020;37(1):86–92. doi: 10.1111/pde.14016 31642120

[51] Abbasi E, Daliri S, Yazdani Z, Mohseni S, Mohammadyan G, Seyed Hosseini SN, et al. Evaluation of resistance of human head lice to pyrethroid insecticides: A meta-analysis study. Heliyon. 2023;9(6):e17219. doi: 10.1016/j.heliyon.2023.e17219 37408932 PMC10319209

[52] Campos EVR, de Oliveira JL, Pascoli M, de Lima R, Fraceto LF. Neem Oil and Crop Protection: From Now to the Future. Front Plant Sci. 2016;7:1494. doi: 10.3389/fpls.2016.01494 27790224 PMC5061770

[53] Brown C, Burgess I. Can neem oil help eliminate lice? Randomized controlled trial with and without louse combing. Adv Pediatr Res. 2017;4(9).

[54] Heukelbach J, Oliveira FAS, Speare R. A new shampoo based on neem (Azadirachta indica) is highly effective against head lice in vitro. Parasitol Res. 2006;99(4):353–6. doi: 10.1007/s00436-006-0146-7 16568334

[55] Candy K, Akhoundi M, Izri A. Pediculicidal activity assessment of four essential oil terpenoids using filter contact and immersion bioassays. Trop Parasitol. 2020;10(2):165–7. doi: 10.4103/tp.TP_41_19 33747889 PMC7951071

[56] Yang Y-C, Lee H-S, Clark JM, Ahn Y-J. Insecticidal activity of plant essential oils against Pediculus humanus capitis (Anoplura: Pediculidae). J Med Entomol. 2004;41(4):699–704. doi: 10.1603/0022-2585-41.4.699 15311463

[57] Toloza AC, Zygadlo J, Biurrun F, Rotman A, Picollo MI. Bioactivity of Argentinean essential oils against permethrin-resistant head lice, Pediculus humanus capitis. J Insect Sci. 2010;10:185. doi: 10.1673/031.010.14145 21062140 PMC3016758

[58] Picollo MI, Toloza AC, Mougabure Cueto G, Zygadlo J, Zerba E. Anticholinesterase and pediculicidal activities of monoterpenoids. Fitoterapia. 2008;79(4):271–8. doi: 10.1016/j.fitote.2008.01.005 18321657

[59] Burgess IF. Physically acting products for head lice–the end of the beginning. PeerJ Preprints. 2018;6.

