# Supplementary material for: Therapeutic potential of plant-based therapies in pediculosis capitis: Systematic review and meta-analysis
Source: PLOS Glob Public Health. 2025 Jul 17;5(7):e0004841. doi: 10.1371/journal.pgph.0004841 (PMC12270178; doi:10.1371/journal.pgph.0004841)
Supplement: S1 Table — (DOCX) [file pgph.0004841.s001.docx]

# **S1 Table: Characteristics of included studies**

Study Methods. No. (n=20)

| **Study Design**  Randomized control trials [1-13]  Non-Randomized control trials [14-20] | 13 (65%)  7 (35%) |
| --- | --- |
| **Country**  Australia [2,4,6]  USA [14]  Middle East (Iran, Iraq, Israel) [1,7,20]  Egypt [5,15-17,19]  Brazil [8]  Europe (Italy, England) [3,10]  Asia (Philippines/Thailand) [9,11-13,18] | 3  1  3  5  1  2  5 |
| **Participants**  **Number of participants in study**  <50 [8,10,12-14,17-20]  50-99 [6,7,16]100-149 [1-5, 15]  >150 [9,11]  **Gender**  Female [12,18,19]  Female and Male [1-9,11,13-17]Not stated [10,20]  **Age Group**  Children only [1,2,4-6,9,12-14,16-19]  Children and adults [3,7,15,20]  Not stated [8,10,11] | 9  3  6  2  3  15  2  13  4  3 |
| **Intervention**  Neem based [5,13,16,18,19]  Anise [3,10]  Eucalyptus oil [2,6]  Others [1,4,7-9,11,12,14,15,17,20] | 5  2  2  11 |
| **Frequency of application**  1x [8,12,17,19,20]  2x every 7 to 9 days [3,5,9,11,16,18]  3x every 5-10 days [1,2,4,6,10,13,14]  Daily for 3 days [15]  6x daily for 3 days repeated in 7 days [7] | 5  6  7  1  1 |
| **Methods of detection**  Nit comb only [1,3,5,7-13,17,18, 19]  Wet combing with nit comb [2,4,6]  Visual inspection only [7,14,15,20]  Not stated [16] | 15  3  4  1 |
| **Outcomes:**  Initial cure rate (1-7 days after first treatment) [3,6,7,8,10]  Final cure rate (defined as no lice/1-14 days after final treatment) [1-10]  Adverse effects [1-14,16-18] | 4  10  17 |

**References:**

1. Mumcuoglu KY, Miller J, Zamir C, Zentner G, Helbin V, Ingber A. The in vivo pediculicidal efficacy of a natural remedy. Isr Med Assoc J. 2002;4(10):790-3.
2. Greive KA, Barnes TM. The efficacy of Australian essential oils for the treatment of head lice infestation in children: A randomised controlled trial. Aust. J. Dermatol. 2017;59(2):e99-e105.
3. Burgess IF, Brunton ER, Burgess NA. Clinical trial showing superiority of a coconut and anise spray over permethrin 0.43% lotion for head louse infestation, ISRCTN96469780. Eur. J. Paediatr. 2010;169(1):55-62.
4. Barker SC, Altman PM. A randomised, assessor blind, parallel group comparative efficacy trial of three products for the treatment of head lice in children-melaleuca oil and lavender oil, pyrethrins and piperonyl butoxide, and a. BMC Dermatol. 2010;10(1):1-7.
5. Semmler M, Abdel-Ghaffar F, Gestmann F, et al. Randomized, investigator-blinded, controlled clinical study with lice shampoo (Licener) versus dimethicone (Jacutin® Pedicul Fluid) for the treatment of infestations with head lice. Parasitol. Res. 2017;116:1863-1870.
6. Grieve K, Altman P, Rowe S, Staton J, Oppenheim V. A randomised, double-blind, comparative efficacy trial of three head lice treatment options: malathion, pyrethrins with piperonyl butoxide and MOOV Head Lice Solution. Aust Pharm. 2007;26(9):738-743.
7. Maarefvand M, Kenari HM, Ghobadi A, Soleymani A, Hashem-Dabaghian FH-D. Efficacy of the Peganum Harmala Oil Versus 1% Permethrin Shampoo on the Treatment of Head Louse Infestation. J.Pharm. Res. Int. 2019;27(6):1-9.
8. Cardoso JH, Noronha Coelho de Souza A, Militão de Souza F, Sa Preire S, Pinçon C. Treatment of Head Louse Infestation with a Novel Mixture Made of Semi-Crystalline Polymers and Plant Extracts: Blind, Randomized, Controlled, Superiority Trial. Cosmetics. 2020;7(2):25.
9. Moreno-Alsalsua M. Randomized controlled trial on the effect of coconut oil, vinegar plus cooking coconut oil versus 1% permethrin shampoo in the treatment of pediculosis. Ped Infect Dis Soc Phil J. 2016;17(2):4-13. .
10. Scanni G, Bonifazi E. Efficacy of a single application of a new natural lice removal product. Preliminary data. Eur. J. Pediatr. 2006;16(4):231.
11. Soonwera M. Efficacy of herbal shampoo base on native plant against head lice (Pediculus humanus capitis De Geer, Pediculidae: Phthiraptera) in vitro and in vivo in Thailand. Parasitol Res. 2014;113(9):3241-3250.
12. Tiangda C, Gritsanapan W, Sookvanichsilp N, Limchalearn A. Anti-headlice activity of a preparation of Annona squamosa seed extract. Southeast Asian J Trop Med. Public Health. 2000;31:174-177.
13. Sabellina LAN, Salamanca CSS, Santos DEA, et al. Effectiveness of neem seed oil methanolic extract shampoo versus permethrin shampoo in the reduction of head lice infestation in children. UERM Health Sci J. 2018:58.
14. McCage C, Ward S, Paling C, Fisher D, Flynn P, McLaughlin J. Development of a paw paw herbal shampoo for the removal of head lice. Phytomed. 2002;9(8):743-748.
15. El-Basheir ZM, Fouad M. A preliminary pilot survey on head lice, pediculosis in Sharkia Governorate and treatment of lice with natural plant extracts. J. Egypt. Soc. Parasitol. 2002;32(3):725-736.
16. Abdel-Ghaffar F, Semmler M. Efficacy of neem seed extract shampoo on head lice of naturally infected humans in Egypt.Parasitol. Res. 2007;100:329-332.
17. Abdel-Ghaffar F, Semmler M, Al-Rasheid K, Klimpel S, Mehlhorn H. Efficacy of a grapefruit extract on head lice: a clinical trial. Parasitol. Res. 2010;106:445-449.
18. Thawornchaisit P, Amornsak W, Mahannop P, et al. Combined neem oil 6% w/w and eucalyptus oil 16% w/w lotion for treating head lice: in vitro and in vivo efficacy studies. J Pharm Pract Res. 2012;42(3):189-192
19. Abdel-Ghaffar F, Al-Quraishy S, Al-Rasheid KA, Mehlhorn H. Efficacy of a single treatment of head lice with a neem seed extract: an in vivo and in vitro study on nits and motile stages. Parasitol Res. 2012;110:277-280.
20. Al-Zayyadi SW. Study of the effectiveness of some raw plants and materials in the treatment of pediculosis in Najaf Province Iraq. Indian J Med Forensic Med  Toxicol. 2020;14(1):499-503.
