# Supplementary material for: Therapeutic potential of plant-based therapies in pediculosis capitis: Systematic review and meta-analysis
Source: PLOS Glob Public Health. 2025 Jul 17;5(7):e0004841. doi: 10.1371/journal.pgph.0004841 (PMC12270178; doi:10.1371/journal.pgph.0004841)
Supplement: S2 Table — (DOCX) [file pgph.0004841.s002.docx]

| Author (Year) | Cardoso et al (2020) |
| --- | --- |
| Study design | Randomized control trial, double blinded |
| Funding | Nil |
| Participants | 45 |
| Country | Brazil |
| Gender | 7 Males, 38 Females |
| Age | >3y |
| Medicinal Plants  Plants (Scientific)  Plant (Common)  Country where native plants originates  Part of plant | *Protium hepatophyllum*  Protium hepatophyllum; in grape-seed oil and Citrus aurantium  Brazil  Resin extract |
| Intervention | Resin extract of *P. hepatophyllum* in grape seed oil, orange peel wax, surfactants (Eur-Amid N2) |
| Dose duration, frequency | 1 treatment for 15min |
| Comparator | Permethrin 1% shampoo |
| Dose duration, frequency | 1 treatment for 15 minutes |
| Mechanism of action | Eur-Amid N2 and phytosterols help to dissolve the epicuticular waxes covering the shells of the parasites. The resin then hardens and causes cracking of the shell of the parasites resulting in breakdown of the external defences and normal biological function. |
| Detection method | Fine head louse comb |
| Final time point measurement | D7 post 1 treatment (D8) |
| Intervention rate (%) | 21/22 (95.4%) |
| Comparator rate (%), p-value | 9/23 (39.1%), p<0.0001 |
| Adverse events | None |

| Author (Year) | Maarefvand et al (2019) |
| --- | --- |
| Study design | Randomized controlled trial, non-blinded |
| Funding | Nil |
| Participants | 93 |
| Country | Iran |
| Gender | 11Males, 82 Females |
| Age | 2y-52 yr |
| Medicinal Plants  Plants (Scientific)  Plant (Common)  Country where native plants originates  Part of plant | *Perganum harmala L*  Perganum harmala oil  Iran  Essential oil |
| Intervention | Perganum harmala oil |
| Dose duration, frequency | 6 treatments for 20minutes (D1,2,3 and 8,9,10) |
| Comparator | Permethrin shampoo |
| Dose duration, frequency | 2 treatments for unknown duration  (D1 and D10) |
| Mechanism of action | Dries moisture and acts as a resolvant |
| Detection method | Direct examination |
| Final time point measurement | D1 post 6 treatments (D11) |
| Intervention rate (%) | 44/48 (91.7%) |
| Comparator rate (%), p-value | 39/45 (86.7%), p<0.005 |
| Adverse events | Perganum harmala oil: Irritation^a^ /Headache (1)  Permethrin: Irritation^a^ (2), Headache (1) |

| Author (Year) | Sabellina et al (2018)^b^ |
| --- | --- |
| Study design | Randomized control trial, single blinded  single blind non inferiority randomized control trial^c^ |
| Funding | Nil |
| Participants | 30 |
| Country | Phillipines |
| Gender | 5 Males, 25 Females |
| Age | 6-14y |
| Medicinal Plants  Plants (Scientific)  Plant (Common)  Country where native plants originates  Part of plant | *Azadirachta indica*  Neem  East India/Burma  Oil/ Methanolic extract, azadirachtin- complex tetranorterpenoid limonoid, nimbin and salannin |
| Intervention | Neem seed oil 10% methanolic shampoo |
| Dose duration, frequency | 3 treatments for 10-15minutes (D0, D10, D20) |
| Comparator | Permethrin 1% shampoo  Pure shampoo (glycol stearate) |
| Dose duration, frequency | 3 treatments for 10-15minutes (D0, D10, D20) |
| Mechanism of action | Contains azadirachtin, complex limonoid that affects physiologic and behavioural aspects in wide variety of insects. Has other components- nimbin, salannin and azadirachtin that is useful in preventing resistance. |
| Detection method | nit comb |
| Final time point measurement | D0 post 3 treatments (D30) |
| Intervention rate (%) | Mean reduction of headlice count: 17.8+/- 23.97, p=0.043 |
| Comparator rate (%), p-value | Mean reduction of head lice count: Permethrin: 22.5 +/- 23.47, p= 0.013  Pure shampoo: NR |
| Adverse events | None |

| Author (Year) | Semmler et al (2017) |
| --- | --- |
| Study design | Randomized controlled trial, single-blinded |
| Funding | Author Gestmann is employee of sponsor (Alpha Biocare Gmbh Neuss Germany) |
| Participants | 119 |
| Country | Egypt |
| Gender | 31 Males, 88 Females |
| Age | 3y-11 y |
| Medicinal Plants  Plants (Scientific)  Plant (Common)  Country where native plants originates  Part of plant | A*zadirachta indica*  Neem  NE India    seed extract |
| Intervention | Neem shampoo (Licener) |
| Dose duration, frequency | 2 treatments for 10minutes (D1, D9) |
| Comparator | Dimethicone (Jacutin) |
| Dose duration, frequency | 2 treatments for 10 minutes (D1, D9) |
| Mechanism of action | Suffocation |
| Detection method | Lice comb |
| Final time point measurement | D4 post 2 treatments (D13) |
| Intervention rate (%) | 60/60 (100%) |
| Comparator rate (%), p-value | 52/54 (96.2%,), p=0.0024 |
| Adverse events | No adverse or serious events. In most cases, the rigorous combing was felt unpleasant. |

| Author (Year) | Greive and Barnes, (2017) |
| --- | --- |
| Study design | Randomized controlled trial, single-blinded |
| Funding | Ego Pharmaceuticals/ manufacturer of MOOV Head Lice solution |
| Participants | 97 |
| Country | Australia |
| Gender | Males and females |
| Age | Primary school children |
| Medicinal Plants  Plants (Scientific)  Plant (Common)  Country where native plants originates  Part of plant | NA  *Eucalyptus oil and Leptospermum petersonii*  Australia  essential oil |
| Intervention | Eucalyptus oil 11% and Leptospermum petersonii 1% (MOOV HeadLice solution) |
| Dose duration, frequency | 3 treatments for 10minutes (D0, D7, D21) |
| Comparator | Pyrethrin 1.65mg/g/ Piperonyl Butoxide 16.5 mg/g (Banlice Mousse) |
| Dose duration, frequency | 2 treatments for 10 minutes(D0, D7) |
| Mechanism of action | NR |
| Detection method | Wet combing |
| Final time point measurement | D1 post 3 treatments(D21) |
| Intervention rate (%) | 33/40 (82.5%) |
| Comparator rate (%), p-value | 13/36 (36.1%), p<0.001 |
| Adverse events | Moov : Irritation^a^ (18)  BanLice Mousse: Irritation^a^ (3) |

| Author (Year) | Greive and Altman (2007) |
| --- | --- |
| Study design | Randomized control trial, double blinded |
| Funding | Ego Pharmaceuticals |
| Participants | 113 |
| Country | Australia |
| Gender | Males and females |
| Age | Primary school children |
| Medicinal Plants  Plants (Scientific)  Plant (Common)  Country where native plants originates  Part of plant | *Eucalyptus, Leptospermum petersonii*  Eucalyptus oil  Australia    essential oil |
| Intervention | Eucalyptus oil 11%, Leptospermum petersonii 1% (MOOV HeadLice Solution) |
| Dose duration, frequency | 3 treatments for 10minutes (D0, D7, D14) |
| Comparator | 16.5mg/g Piperonyl butoxide and 1.65mg/g pyrethrin (BanLice Mousse)  1% malathion (KP24) |
| Dose duration, frequency | 2 treatments for 10 minutes (BanLice) or 30 minutes (KP24)  (D0, D7) |
| Mechanism of action | NR |
| Detection method | Wet combing |
| Final time point measurement | D7 post 3 treatments (D21) |
| Intervention rate (%) | 33/40 (82.5%) |
| Comparator rate (%), p-value | Banlice 13/36 (36.1%), p<0.0001  KP24 11/37 (26.7%), p<0.0001 |
| Adverse events | Moov Headlice Solution: Irritation (18)  Banlice: Irritation^a^ (3)  KP: Irritation^a^ (2) |

| Author (Year) | Mumcuoglu et al,(2002) |
| --- | --- |
| Study design | Randomized control trial, non-blinded |
| Funding | NR |
| Participants | 143 |
| Country | Israel |
| Gender | Males and females |
| Age | 6-14yrs |
| Medicinal Plants  Plants (Scientific)  Plant (Common)  Country where native plants originates  Part of plant | *Cananga odorata, Cananga odorata, Cocos nucifera, Pimpinella anisum*  coconut and anise  Israel  NA |
| Intervention | Coconut, anise and ylang ylang oils (Chick-chack spray) |
| Dose duration, frequency | 3 treatments for 15 minutes (D1, D5, D10) |
| Comparator | Permethrin 0.5%, Malathion 0.25%, Piperonyl butoxide 2%, Isododecane 47.25% and propellant gas 50% (Paraplus) |
| Dose duration, frequency | 2 treatments for 10 minutes (D1, D10) |
| Mechanism of action | NR |
| Detection method | Louse comb |
| Final time point measurement | D0 post 3 treatments (D10) |
| Intervention rate (%) | 60/70 (85.7%) |
| Comparator rate (%), p-value | 56/73 (56.7%), p>0.05 |
| Adverse events | Chick Chack Spray : Irritation^a^ (1), Odour (5)  Paraplus: Irritation^a^ (1), Odour (4) |

| Author (Year) | Moreno-Alsasua (2016) |
| --- | --- |
| Study design | Randomized controlled trial, single-blinded |
| Funding | Nil |
| Participants | 150 |
| Country | Philippines |
| Gender | 11 Males, 139 Females |
| Age | 3 - 12yrs |
| Medicinal Plants  Plants (Scientific)  Plant (Common)  Country where native plants originates  Part of plant | *Cocos nucifera, acetic acid*  Coconut oil and vinegar (CV) OR coconut oil (CO)  SE Asia  Oil |
| Intervention | Coconut oil and vinegar or coconut oil |
| Dose duration, frequency | 2 treatments for 8hours(D1, D8) |
| Comparator | Permethrin 1% shampoo |
| Dose duration, frequency | 2 treatment for 5 minutes (D1, D8) |
| Mechanism of action | Suffocation of respiratory spiracles of headlice and eggs by the oil |
| Detection method | Nit comb |
| Final time point measurement | D6 post 2 treatments (D14) |
| Intervention rate (%) | CO 32/50 (64%)  CV 47/50 (94%) |
| Comparator rate (%), p-value | 49/50 (98%, p=0.00) |
| Adverse events | Coconut oil: None  Coconut oil and vinegar: None  Permethrin: Eye irritation(8) , Irritation^a^ (3) |

| Author (Year) | Burgess et al (2009) |
| --- | --- |
| Study design | Randomized controlled trial, single-blinded |
| Funding | Mega Pharma NV, Nazareth, Belgium |
| Participants | 100 |
| Country | England |
| Gender | 20 Males, 80Females |
| Age | 2y - 49y |
| Medicinal Plants  Plants (Scientific)  Plant (Common)  Country where native plants originates  Part of plant | *Cocos nucifera and Pimpinella anisum*  Coconut and anise  NR  essential oil |
| Intervention | Fractionated coconut oil (caprylic capric triglyceride), propan-1-ol, anise oil (from star anise), and ylang-ylang flower oil spray |
| Dose duration, frequency | 2 treatments for 15 minutes (D1, D9) |
| Comparator | Permethrin 0.43% spray |
| Dose duration, frequency | 2 treatments for 45 minutes (D1, D9) |
| Mechanism of action | Coats head lice in oily film obstructing respiratory system |
| Detection method | Dry detection comb |
| Final time point measurement | D5 post 2 treatments (D14) |
| Intervention rate (%) | 46/50 (92.0%) |
| Comparator rate (%), p-value | 24/48 (50%), p<0.0001 |
| Adverse events | Fractionated coconut oil (caprylic capric triglyceride), propan-1-ol, anise oil (from star anise), and ylang-ylang flower oil spray: Irritation^a^ (17)  Permethrin: Irritation^a^ (20) |

| Author (Year) | Scanni (2005) |
| --- | --- |
| Study design | Randomized control trial |
| Funding | Nil |
| Participants | 24 |
| Country | Italy |
| Gender | NR |
| Age | 4-15y |
| Medicinal Plants  Plants (Scientific)  Plant (Common)  Country where native plants originates  Part of plant | *Cananga odorata*, *Cocos nucifera*, *Pimpinella anisum*  Coconut oil extract, anise, ylang ylang oil (Paranix)  NR  essential oil |
| Intervention | Coconut oil, anise and ylang ylang oil spray (Paranix) |
| Dose duration, frequency | 3 treatments for 15minutes (D0, D6, D11) |
| Comparator | Malathion |
| Dose duration, frequency | 2 treatments for 10minutes (D0, D7) |
| Mechanism of action | NR |
| Detection method | Metal comb |
| Final time point measurement | D1 post 3 treatments (D0, 6, 11) |
| Intervention rate (%) | 11/11 (100%) |
| Comparator rate (%), p-value | 11/11 (100%), p= NR |
| Adverse events | Paranix: Odour (1)  Malathion: Odour (2) |

| Author (Year) | Soonwera (2014)^b^ |
| --- | --- |
| Study design | Randomized control trial, single blinded |
| Funding | Faculty of Agricultural Technology, King Mongkut's Institute of Technology Lakdrabang, Thailand, Bangkok. |
| Participants | 210 |
| Country | Thailand |
| Gender | Males and females |
| Age | 3-12y |
| Medicinal Plants  Plants (Scientific)  Plant (Common)  Country where native plants originates  Part of plant | *Acorus calamus Linn., Phyllanthus emblica Linn., Zanthoxylum limonella Alston.*  Acorus calamus shampoo, phyllanths emblica shampoo, zanthoxylum limonella  Thailand  Rhizome, Fruit and Fruit |
| Intervention | 10% w/v crude extract of *Acorus calamus* rhizomes., *Phyllanthus emblica*. fruits, *Zanthoxylum limonella* fruits |
| Dose duration, frequency | 2 treatments for 15minutes (D1, D7) |
| Comparator | Malathion shampoo 1% shampoo  Carbaryl 0.6% shampoo  Babi Mild Natural N Mild shampoo  Johnson's baby shampoo |
| Dose duration, frequency | 2 treatments for 15 minutes (D1, D7) |
| Mechanism of action | NR |
| Detection method | Fine toothed comb |
| Final time point measurement | D1 post 2 treatments (7d) |
| Intervention rate (%) | Absolute rate NR  (100%) |
| Comparator rate (%), p-value | Absolute rate NR  Malathion shampoo (85.33%), p<0.05  Carbaryl shampoo 93%,  Babi Mild Natural N Mild shampoo (0%), p<0.05  Johnson‘s Baby shampoo (0%), p<0.05 |
| Adverse events | 10% w/v crude extract of Acrous calamus rhizomes, Phyllanthus emblica, fruits, Zanthoxylum limonella fruits: None  Carbaryl and malathion shampoo: Irritation Present but NR no. events) |

| Author (Year) | Barker & Altman (2010) |
| --- | --- |
| Study design | Randomized controlled trial, single-blinded |
| Funding | Key Pharmaceuticals (No involvement in design, interpretation, manuscript) |
| Participants | 123 |
| Country | Australia |
| Gender | Males and females |
| Age | 4y - 12y |
| Medicinal Plants  Plants (Scientific)  Plant (Common)  Country where native plants originates  Part of plant | *Melaleuca alterniflora and Lavandula*  Tea tree oil and lavender oil  Australia  essential oil |
| Intervention | Tea tree oil 10% and lavendar oil 1% (NeutraLice Lotion) |
| Dose duration, frequency | 3 treatments (D0, D7, D14) |
| Comparator | Pyrethin 1.65mg/g/ piperonyl butoxide 16.5mg/g (BanLice Mousse) |
| Dose duration, frequency | 2 treatments for 10 minutes (D0, D7) |
| Mechanism of action | Suffocation |
| Detection method | Wet combing |
| Final time point measurement | D1 post 3 treatments (D15) |
| Intervention rate (%) | 41/42 (97.6%) |
| Comparator rate (%), p-value | 10/40 (25.0%), p<0.0001 |
| Adverse events | NeutraLice Lotion: Irritation^a^ (29)  BanLice Mousse: Irritation^a^ (4) |

| Author (Year) | Tiangda (2000)^b^ |
| --- | --- |
| Study design | Randomized control trial, non-blinded |
| Funding | Nil |
| Participants | 22 |
| Country | Thailand |
| Gender | Females |
| Age | 6-10y |
| Medicinal Plants  Plants (Scientific)  Plant (Common)  Country where native plants originates  Part of plant | *Annosa squamosa Linn.*  Custard apple  Tropical Americas and West Indies  Organic solvent (petroleum) extract of seeds |
| Intervention | Custard apple seed extract 20% w/w oil in water cream |
| Dose duration, frequency | 1 treatment for 3hours |
| Comparator | Control (cream base)  Benzyl benzoate 25% emulsion with fine combing |
| Dose duration, frequency | 1 treatment for 3hours |
| Mechanism of action | NR |
| Detection method | Fine comb |
| Final time point measurement | D0 post 1 treatment |
| Intervention rate (%) | Proportion of dead lice: 261/274 (95.3%) |
| Comparator rate (%), p-value | Proportion of dead lice: Cream base 31/73 (47.4%, p<0.05), Benzyl benzoate 15/33 (60.1%, p<0.05) |
| Adverse events | Custard apple seed extract 20% w/w oil in water cream: None  Benzyl benzoate 25% emulsion: Irritation present but NR no. events |

NR- not reported

a: Irritation: itch, stinging and/or burning

b. Not included in quantitative analysis as no cure rates reported in studies.

c: Groups do not compare neem vs no neem but rather combing vs combing with placebo comb
