# Supplementary material for: Therapeutic potential of plant-based therapies in pediculosis capitis: Systematic review and meta-analysis
Source: PLOS Glob Public Health. 2025 Jul 17;5(7):e0004841. doi: 10.1371/journal.pgph.0004841 (PMC12270178; doi:10.1371/journal.pgph.0004841)
Supplement: S3 Table — (DOCX) [file pgph.0004841.s003.docx]

| Author (Year) | Al Zayadi (2020) |
| --- | --- |
| Study design | Single arm trial |
| Funding | Self funded |
| Participants | 45 |
| Country | Iraq |
| Gender | NR |
| Age | 8-30y |
| Medicinal Plants  Plants (Scientific)  Plant (Common)  Country where native plants originates  Part of plant | *Lawsonia inermis, allium cepa, curcuma longa*  Henna paste, onion, Curcumin  NR  Lawsonia- leaves, allium cepa- purple onion juice- curcuma longa-ground turmeric stalks |
| Intervention | Group 1:110g Lawsonia leaves with 200 ml water+ 5g mercury  Group 2: Onion juice 150g + 10g ground turmeric  Group 3: 250 mls vinegar+ 25g salt |
| Dose duration, frequency | 1 treatment for 2 hours |
| Comparator | NA |
| Dose duration, frequency | NA |
| Mechanism of action | Onion contains sulfur and flavonoids which is antimicrobial and causes immediate and total inhibition of DNA and RNA and protein synthesis, as well as contains essential oil that prevent lice respiration. Mercury is a chemical toxin that affects nervous system. Vinegar softens the protective sheath of lice. Sodium chloride mechanism of action is not fully known but potentially causes a tear in the digestive system |
| Detection method | Direct examination |
| Final time point measurement | D0 post 1 treatment (2 hours post treatment) |
| Intervention rate(%) | Lawsonia and mercury :15/15 100%)  Onion juice and turmeric :12/15 (80%)  Vinegar and sodium chloride: 7/15 (46.7%) |
| Comparator rate (%) | None |
| Adverse events | NR |

| Author (Year) | Thawornchaisit et al (2012) |
| --- | --- |
| Study design | Single arm trial |
| Funding | co-sponsored by Biomimic Pty Ltd (Brisbane) and Pharmacare Laboratories Pty Ltd (Sydney) |
| Participants | 45 |
| Country | Thailand |
| Gender | Females |
| Age | 7-12y |
| Medicinal Plants  Plants (Scientific)  Plant (Common)  Country where native plants originates  Part of plant | *Azadirachta indica + Eucalyptus*  Neem oil+ eucalyptus oil  North East India  Oil from seed of neem tree |
| Intervention | Neem oil 6% and eucalyptus oil 16% in hydrophobic carrier (lanolin+ silicone) |
| Dose duration, frequency | 2 treatments for 30 minutes (D0, D7) |
| Comparator | None |
| Dose duration, frequency | None |
| Mechanism of action | NR |
| Detection method | Fine toothed louse comb |
| Final time point measurement | D7 post 2 treatments (14d) |
| Intervention rate (%) | Absolute rate NR (89%) |
| Comparator rate (%) | NA |
| Adverse events | Allergic contact dermatitis (1) |

| Author (Year) | Abdel-Ghaffar et al (2012) |
| --- | --- |
| Study design | Single arm trial |
| Funding | Supported by Center of Excellence of College of Science of the King Saud University, Riyadh, Saudi Arabia |
| Participants | 20 |
| Country | Egypt |
| Gender | Females |
| Age | 3-12 y |
| Medicinal Plants  Plants (Scientific)  Plant (Common)  Country where native plants originates  Part of plant | *Azadirachta indica*  Neem  India  Neem Seed extract |
| Intervention | Licener shampoo |
| Dose duration, frequency | 1 treatment for 10 or 20 minutes  12 children treated for 10 mins, 8 children treated for 20mins |
| Comparator | NA |
| Dose duration, frequency | NA |
| Mechanism of action | Physical. Blocks airways |
| Detection method | Fine toothed comb |
| Final time point measurement | D7 post 1 treatment (7 d) |
| Intervention rate(%) | 20/20(100%) |
| Comparator rate (%) | NA |
| Adverse events | NR |

| Author (Year) | Abdel-Ghaffar and Semmler (2006) |
| --- | --- |
| Study design | Single arm trial |
| Funding | NR |
| Participants | 60 |
| Country | Egypt |
| Gender | 23 Males, 37 Females |
| Age | 5 - 15y |
| Medicinal Plants  Plants (Scientific)  Plant (Common)  Country where native plants originates  Part of plant | *Azadirachta indica*  Neem  Egypt  Seed extract |
| Intervention | Neem with variable duration (3 arms)*  1^st^ trial: 7 children were applied for 30 minutes. In the second week, another 10 children were included  2^nd^ trial: 25 children with unknown duration  3^rd^ trial:  6 children in Group 1: 15 minutes  6 children in Group 2 for 10 minutes  6 children in Group 3 for 5 minutes |
| Dose duration, frequency | 1 -2 treatments for 5-30minutes (D1, D10)* |
| Comparator | NA |
| Dose duration, frequency | NA |
| Mechanism of action | NR |
| Detection method | Combing (not specified type) |
| Final time point measurement | D0 post 2 treatments (10d) |
| Intervention rate (%) | Absolute Rate NR (86 - 97%)  1^st^ trial: After 30 minutes, more than 95% of lice were dead. Re-examination at 2weeks showed 87% free of any infestation.  2^nd^ trial: 97% of the 25 children were head lice free  3^rd^ trial: After 10-15 minutes of exposure in Group 1 and 2, 96% of different lice stages were dead, in group 3, 12% of discarded lice were still alive. Re-examination at 1 and 2 weeks after the initial 10-15 minutes treatment were free for any reinfestation apart from 1 case with ‘very fatty hair’. |
| Comparator rate (%) | NA |
| Adverse events | Irritation* |

| Author (Year) | Abdel-Ghaffar et al (2009) |
| --- | --- |
| Study design | Non-Randomised control trial |
| Funding | Partially supported by Centre of Excellence of the College of Science of the King Saud University at Riyadh, Saudi Arabia |
| Participants | 20 |
| Country | Egypt |
| Gender | 4 Males, 16 Females |
| Age | 2-9y |
| Medicinal Plants  Plants (Scientific)  Plant (Common)  Country where native plants originates  Part of plant | *Citrus × paradisi*  Grapefruit extracts  NA  NR |
| Intervention | One group of ten children was exposed for 10 minutes to the product before washing the hair with tap water and subsequent combing. 8 children were exposed for 20 minutes to the product before washing and combing |
| Dose duration, frequency | 1 treatment for 10 minutes or 20 minutes |
| Comparator | 2 children had been washed with pure tap water |
| Dose duration, frequency | NR |
| Mechanism of action | Physical penetration into the tracheal which causes reduction in the transmission of oxygen |
| Detection method | Louse comb by mothers |
| Final time point measurement | D0 post 1 treatment |
| Intervention rate(%) | 18/18 (100%) |
| Comparator rate(%) | 0/2 (0%) |
| Adverse events | None |

| Author (Year) | McCage et al (2002) |
| --- | --- |
| Study design | Single arm trial |
| Funding | Nature's Sunshine Products, Provo, UT |
| Participants | 16 (Group D)-optimal treatment |
| Country | USA |
| Gender | Group A-C, 8 Males ,14Females, 4 NR  Group D: 3 Males, 13 Females |
| Age | Elementary and middle school |
| Medicinal Plants  Plants (Scientific)  Plant (Common)  Country where native plants originates  Part of plant | *Asimia triloba Dunal, Thymus vulgaris, Melaleuca alternifolia (Maiden and Betche) Cheel*  Paw paw tree, thymol and tea tree oil  NR  Paw paw tree extract (twigs), tea tree oil |
| Intervention | Paw paw, Thymol, 1-terpin-4-ol in TTO) in shampoo  Group A: 1% TTO, 0.8% thymol, 0.2% paw paw extract, 98% shampoo  Group B: 0.5% TTO, 1.5% thymol, 1% paw paw extract, 98% shampoo  Group C: 0.5% TTO, 1% thymol, 0.5% paw paw extract, 98% shampoo  (shampoo base prepared by Arizona Naturals, AZ)  Group D: 0.5% TTO, 1% thymol, 0.5% paw paw extract, 98% shampoo (shampoo base prepared by Wasatch Labarotories, Sandy UT) |
| Dose duration, frequency | 3 treatments for 1 hour (D0, D7, D14) |
| Comparator | NA |
| Dose duration, frequency | NA |
| Mechanism of action | Deplete ATP, which can thwart ATP dependent pesticide resistance |
| Detection method | Inspection by nurses |
| Final time point measurement | DX post 3 treatments (21d) |
| Intervention rate (%) | Varying results.  Group A: 1/4 (25%) were lice free but were reinfested.  Group B: 4/4 lice free (100%)  Group C: 18/18 (100%)  Group D: 16/16(100%) |
| Comparator rate (%) | NA |
| Adverse events | Group A and C: NR  Group B: Irritation (5)  Group D: None |

| Author (Year) | El-Bashier and Fouad (2002) |
| --- | --- |
| Study design | Single arm trial |
| Funding | NR |
| Participants | 100 |
| Country | Egypt |
| Gender | 30 Males ,90 Females |
| Age | 3m- > 42 |
| Medicinal Plants  Plants (Scientific)  Plant (Common)  Country where native plants originates  Part of plant | *Lawsonia alba L., Trigonella-faemum-gracanum, Hibiscus cannabinus and Artemisia cina*  Henna, Fenugreek, Hibiscus, Wormseed  Egypt  Henna(leaves) +helba (Trigonella-seeds)/ Henna(leaves)+ karkarde (hibiscus cannabinus-Flowers) + Henna + sheah (Artermisa cina-flowers) |
| Intervention | Henna, helba; or  Henna, karkade; or  Henna, sheah |
| Dose duration, frequency | 3 treatments for 3 hours (between D1 – D7) |
| Comparator | NA |
| Dose duration, frequency | NA |
| Mechanism of action | NR |
| Detection method | Inspection by nurses |
| Final time point measurement | DX post X treatments (7d) |
| Intervention rate (%) | Henna, helba-75/100 (75%)  Henna, karkarde 50/100 (50%)  Henna, sheah 100/100 (100%) |
| Comparator rate (%) | NA |
| Adverse events | NR |

All studies are single-arm trials with exception of Abdel and Ghaffar (2009)

All studies are open label

*Methodology or number of participants involved not clearly defined for treatment duration, dosing, frequency or adverse events

NR- Not reported

NA- Not applicable
