# Supplementary material for: Therapeutic potential of plant-based therapies in pediculosis capitis: Systematic review and meta-analysis
Source: PLOS Glob Public Health. 2025 Jul 17;5(7):e0004841. doi: 10.1371/journal.pgph.0004841 (PMC12270178; doi:10.1371/journal.pgph.0004841)

**PROSPERO Protocol and Summary Table of Amendments Review**

URL: <https://www.crd.york.ac.uk/PROSPERO/view/CRD42023428674>

| **Amendment No.** | **Date** | **Change Description** | **Reason for change** | **Affected section** |
| --- | --- | --- | --- | --- |
| 1.1 | 15^th^ June 2023 | Decision to conduct meta-analysis | To provide quantitative synthesis | Methods |
| 1.2 | 15^th^ August 2024 (post peer review) | Exclusion of study [1] | Minimize bias secondary to potential subtherapeutic concentration | Results |
| 1.3 | 15^th^ August 2024 (post peer review) | Exclusion of study [2] | Study does not investigate primary outcome (uses neem in both intervention and comparator) | Results |
| 1.4 | 15^th^ August 2024  (post peer review) | Exclusion of studies [3,4] | Use of wholly synthetic compounds do not fit inclusion criteria | Methods, results |
| 1.5 | 25^th^ September 2024  (post peer review) | Revision of statistical analysis | Given exclusion of specific studies ^1,3,4^ | Results |

1. Burgess IF, Brunton ER, Burgess NA. Clinical trial showing superiority of a coconut and anise spray over permethrin 0.43% lotion for head louse infestation, ISRCTN96469780. *Eur. J. Paediatr.* 2010;169(1):55-62.
2. Brown C, Burgess I. Can neem oil help eliminate lice? Randomised controlled trial with and without louse combing. *Adv Pediatr Res.* 2017;4(9)
3. Connolly M, Stafford K, Coles G, Kennedy C, Downs A. Control of head lice with a coconut-derived emulsion shampoo. *JEADV*. 2008;23(1):67-69.
4. Burgess IF, Burgess NA. ‘‘Anti-lice Protector Shampoo’’: Clinical Study Shows Lack of Efficacy of Coconut Oil Derivatives in the Elimination of Head Louse Infestation. *Türkiye Parazitol Derg*. 2020;44(4):211.


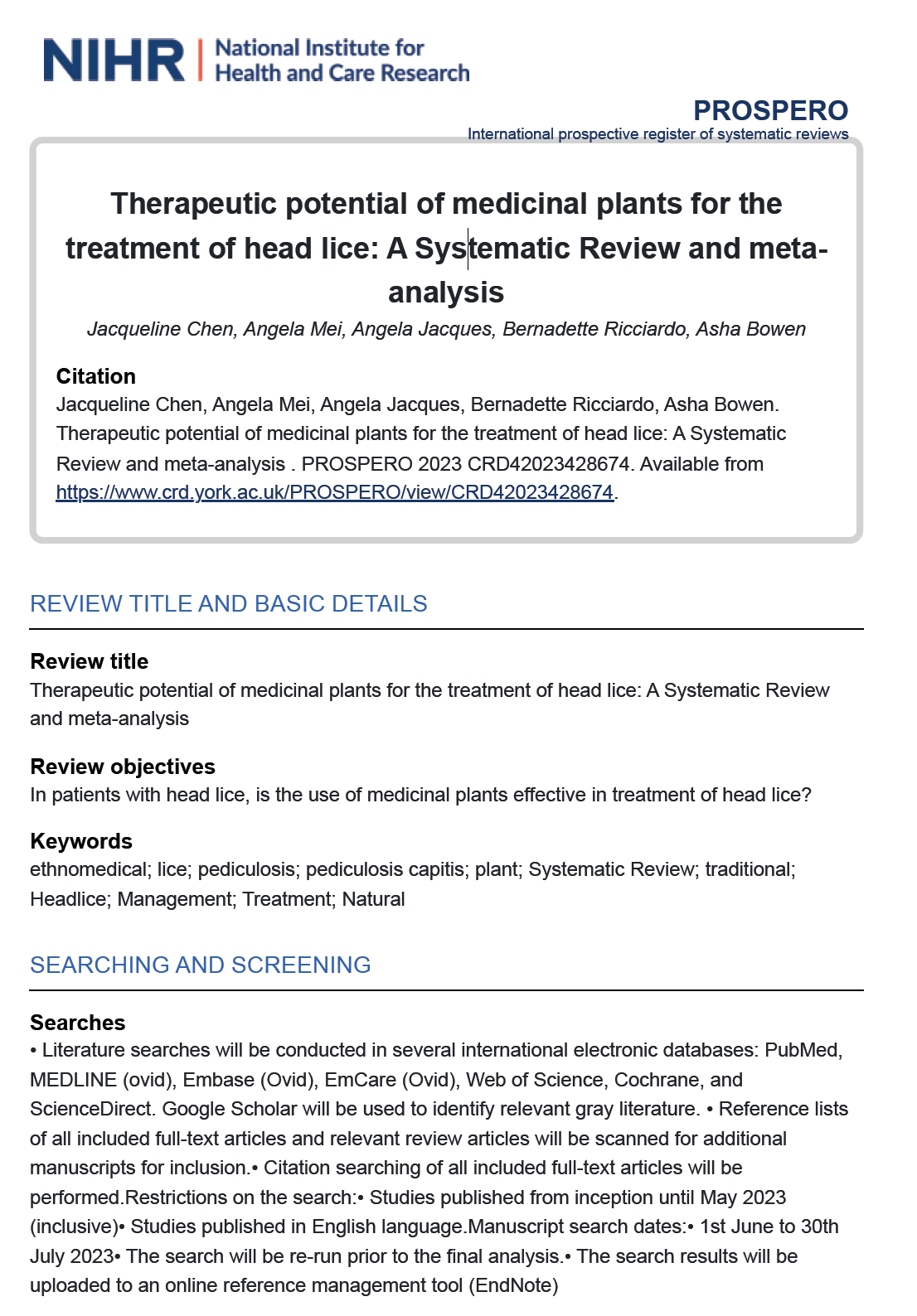


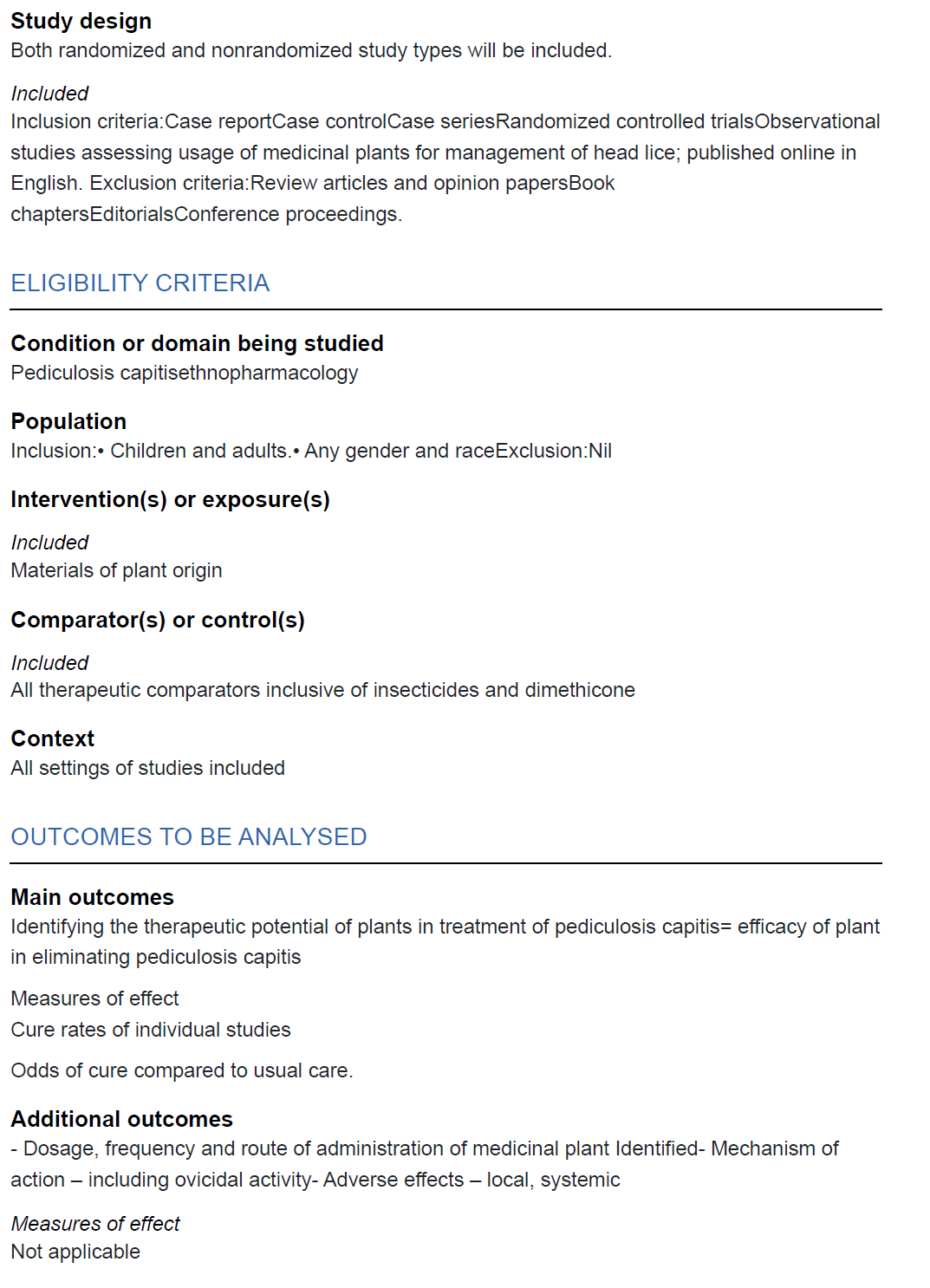


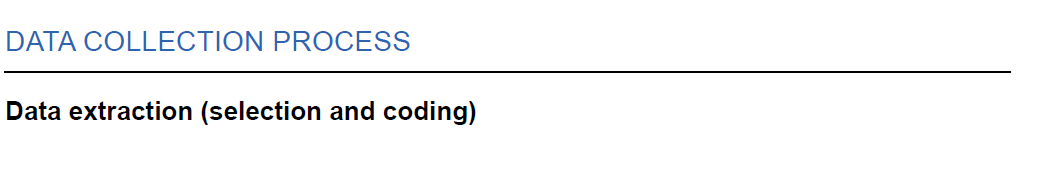


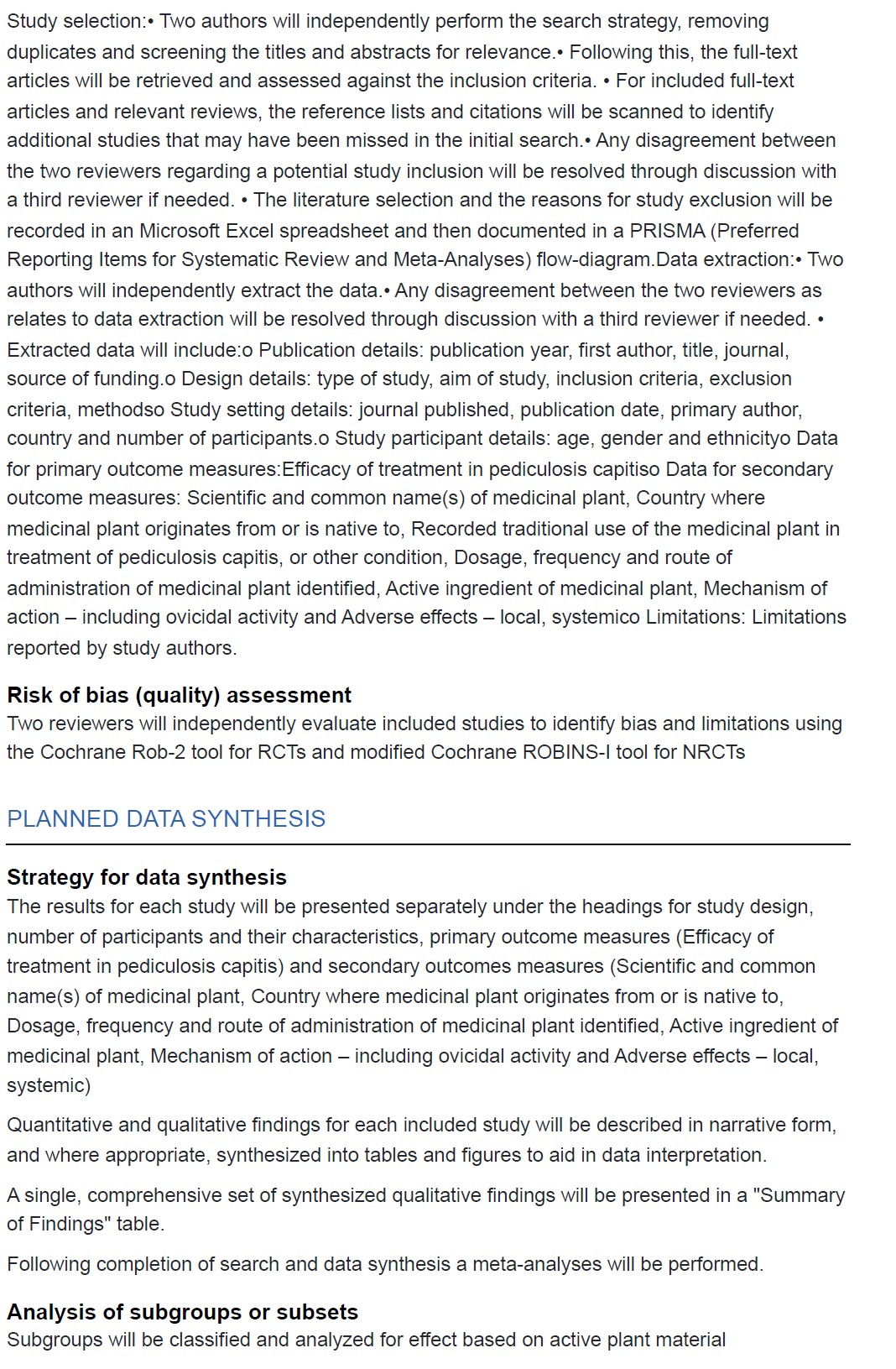


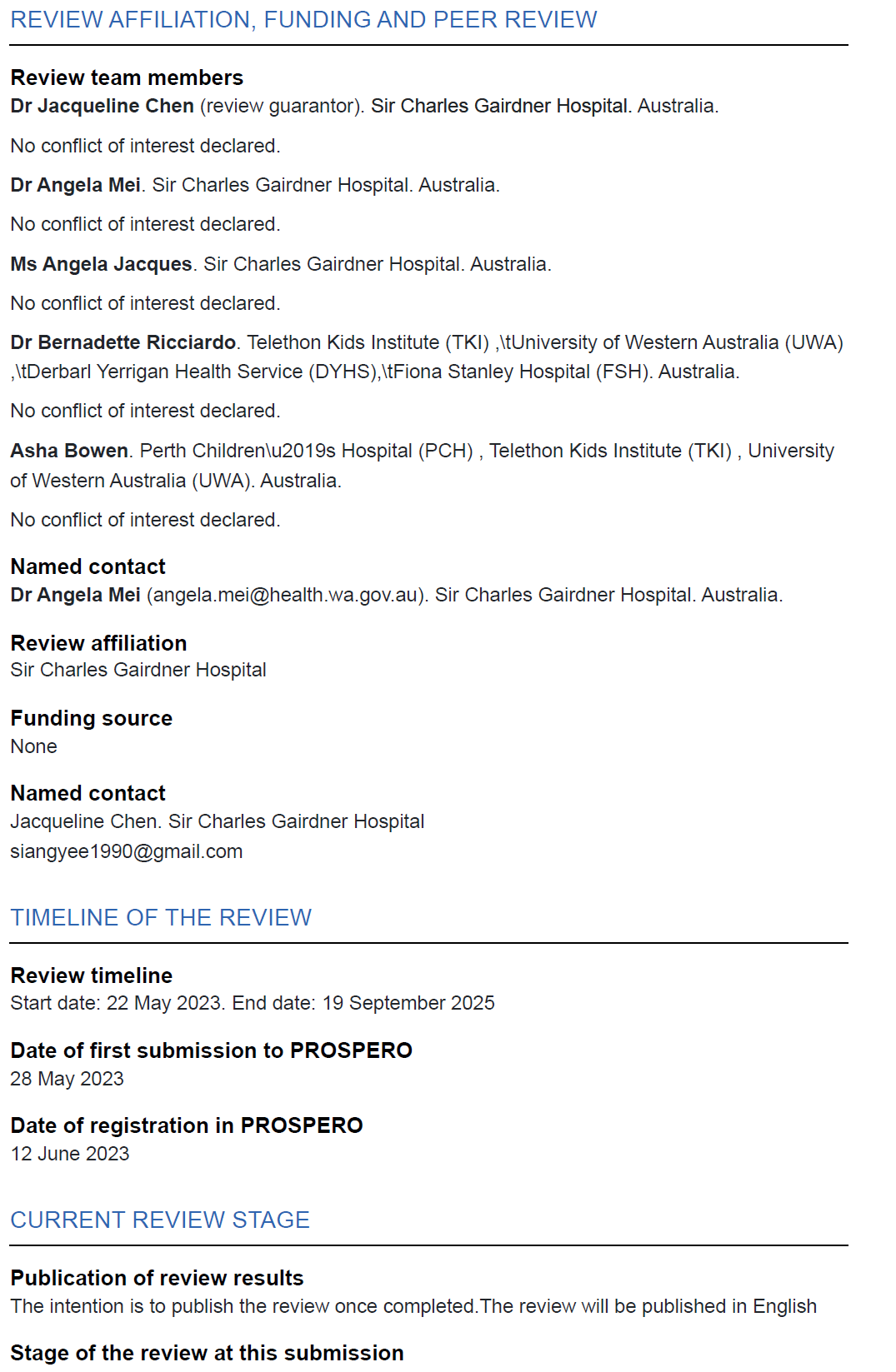


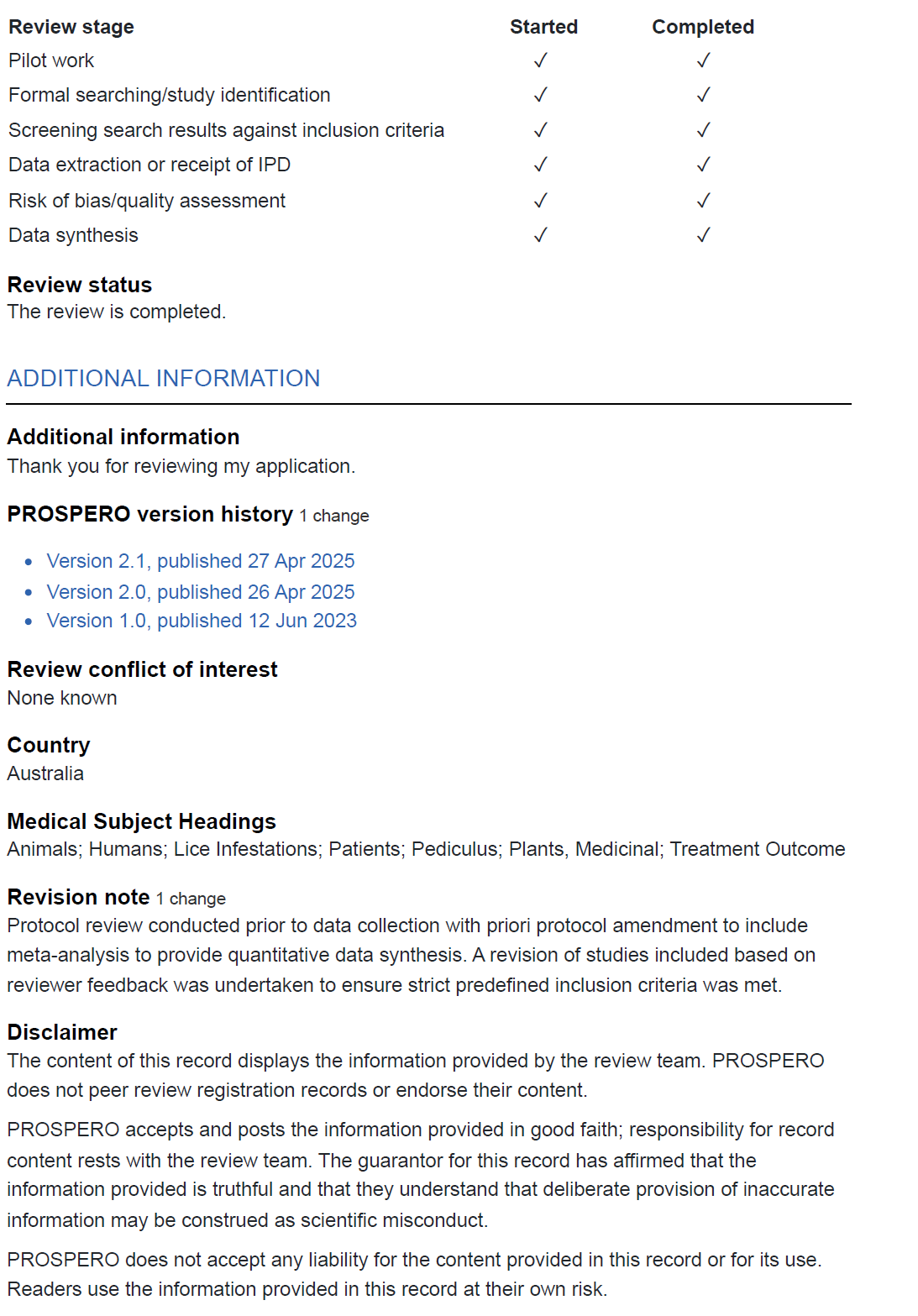

Supplement: S2 File — (DOCX) [file pgph.0004841.s005.docx]
